# EEG-based study of design creativity: a review on research design, experiments, and analysis

**DOI:** 10.3389/fnbeh.2024.1331396

**Published:** 2024-08-01

**Authors:** Morteza Zangeneh Soroush, Yong Zeng

**Affiliations:** Concordia Institute for Information Systems Engineering, Gina Cody School of Engineering and Computer Science, Concordia University, Montreal, QC, Canada

**Keywords:** design creativity, creativity, neurocognition, EEG, higher-order cognitive tasks, thematic analysis

## Abstract

Brain dynamics associated with design creativity tasks are largely unexplored. Despite significant strides, there is a limited understanding of the brain-behavior during design creation tasks. The objective of this paper is to review the concepts of creativity and design creativity as well as their differences, and to explore the brain dynamics associated with design creativity tasks using electroencephalography (EEG) as a neuroimaging tool. The paper aims to provide essential insights for future researchers in the field of design creativity neurocognition. It seeks to examine fundamental studies, present key findings, and initiate a discussion on associated brain dynamics. The review employs thematic analysis and a forward and backward snowball search methodology with specific inclusion and exclusion criteria to select relevant studies. This search strategy ensured a comprehensive review focused on EEG-based creativity and design creativity experiments. Different components of those experiments such as participants, psychometrics, experiment design, and creativity tasks, are reviewed and then discussed. The review identifies that while some studies have converged on specific findings regarding EEG alpha band activity in creativity experiments, there remain inconsistencies in the literature. The paper underscores the need for further research to unravel the interplays between these cognitive processes. This comprehensive review serves as a valuable resource for readers seeking an understanding of current literature, principal discoveries, and areas where knowledge remains incomplete. It highlights both positive and foundational aspects, identifies gaps, and poses lingering questions to guide future research endeavors.

## 1 Introduction

### 1.1 Creativity, design, and design creativity

Investigating design creativity presents significant challenges due to its multifaceted nature, involving nonlinear cognitive processes and various subtasks such as divergent and convergent thinking, perception, memory retrieval, learning, inferring, understanding, and designing (Gero, 1994; Gero, 2011; Nguyen and Zeng, 2012; Jung and Vartanian, 2018; Xie, 2023). Additionally, design creativity tasks are often ambiguous, intricate, and nonlinear, further complicating efforts to understand the underlying mechanisms and the brain dynamics associated with creative design processes.

Creativity, one of the higher-order cognitive processes, is defined as the ability to develop useful, novel, and surprising ideas (Sternberg and Lubart, 1998; Boden, 2004; Runco and Jaeger, 2012; Simonton, 2012). Needless to say, creativity occurs in all parts of social and personal life and all situations and places, including everyday cleverness, the arts, sciences, business, social interaction, and education (Mokyr, 1990; Cropley, 2015b). However, this study particularly focuses on reviewing EEG-based studies of creativity and design creativity tasks.

Design, as a fundamental and widespread human activity, aiming at changing existing situations into desired ones (Simon, 1996), is nonlinear and complex (Zeng, 2001), and lies at the heart of creativity (Guilford, 1959; Gero, 1996; Jung and Vartanian, 2018; Xie, 2023). According to the recursive logic of design (Zeng and Cheng, 1991), a designer intensively interacts with the design problem, design environment (including stakeholders of design, design context, and design knowledge), and design solutions in the recursive environment-based design evolution process (Zeng and Gu, 1999; Zeng, 2004, 2015; Nagai and Gero, 2012). Zeng (2002) conceptualized the design process as an environment-changing process in which the product emerges from the environment, serves the environment, and changes the environment (Zeng, 2015). Convergent and divergent thinking are two primary modes of thinking in the design process, which are involved in analytical, critical, and synthetic processes. Divergent thinking leads to possible solutions, some of which might be creative, to the design problem whereas convergent thinking will evaluate and filter the divergent solutions to choose appropriate and practical ones (Pahl et al., 1988).

Creative design is inherently unpredictable; at times, it may seem implausible – yet it happens. Some argue that a good design process and methodology form the foundation of creative design, while others emphasize the significance of both design methodology and knowledge in fostering creativity. It is noteworthy that different designers may propose varied solutions to the same design problem, and even the same designer might generate diverse design solutions for the same problem over time (Zeng, 2001; Boden, 2004). Creativity may spontaneously emerge even if one does not intend to conduct a creative design, whereas creative design just may not come out no matter how hard one tries. A design is considered routine if it operates within a design space of known and ordinary designs, innovative if it navigates within a defined state space of potential designs but yields different outcomes, and creative if it introduces new variables and structures into the space of potential designs (Gero, 1990). Moreover, it is conceivable that a designer may lack creativity while the product itself demonstrates creative attributes, and conversely, a designer may exhibit creativity while the resulting product does not (Yang et al., 2022).

Several models of design creativity have been proposed in the literature. In some earlier studies, design creativity was addressed as engineering creativity or creative problem-solving (Cropley, 2015b). Used in recent studies (Jia et al., 2021; Jia and Zeng, 2021), the stages of design creativity include problem understanding, idea generation, idea evolution, and idea validation (Guilford, 1959). Problem understanding and idea evaluation are assumed to be convergent cognitive tasks whereas idea generation and idea evolution are considered divergent tasks in design creativity. An earlier model of creative thinking proposed by Wallas (1926) is presented in four phases including preparation, incubation, illumination, and verification (Cropley, 2015b). The “Preparation” phase involves understanding a topic and defining the problem. During “Incubation,” one processes the information, usually subconsciously. In the “Illumination” phase, a solution appears, often unexpectedly. Lastly, “Verification” involves evaluating and implementing the derived solution. In addition to this model, a seven-phase model (an extended version of the 4-phase model) was later introduced containing preparation, activation, generation, illumination, verification, communication, and validation (Cropley, 2015a,b). It is crucial to emphasize that these phases are not strictly sequential or distinct in that interactions, setbacks, restarts, or premature conclusions might occur (Haner, 2005). In contrast to those emperical models of creativity, the nonlinear recursive logic of design creativity was rigorously formalized in a mathematical design creativity theory (Zeng, 2001; Zeng et al., 2004; Zeng and Yao, 2009; Nguyen and Zeng, 2012). For further details on the theories and models of creativity and design creativity, readers are directed to the referenced literature (Gero, 1994, 2011; Kaufman and Sternberg, 2010; Williams et al., 2011; Nagai and Gero, 2012; Cropley (2015b); Jung and Vartanian, 2018; Yang et al., 2022; Xie, 2023).

### 1.2 Design creativity neurocognition

First, we would like to provide the definitions of “design” and “creativity” which can be integrated into the definition of “design creativity.” According to the Cambridge Dictionary, the definition of design is: “to make or draw plans for something.” In addition, the definition of creativity is: “the ability to make something new or imaginative.” So, the definition of design creativity is: “the ability to design something new and valuable.” With these definitions, we focus on design creativity neurocognition in this section.

It is of great importance to study design creativity neurocognition as the brain plays a pivotal role in the cognitive processes underlying design creativity tasks. So, to better investigate design creativity we need to concentrate on brain mechanisms associated with the related cognitive processes. However, the complexity of these tasks has led to a significant gap in our understanding; consequently, our knowledge about the neural activities associated with design creativity remains largely limited and unexplored. To address this gap, a burgeoning field known as design creativity neurocognition has emerged. This field focuses on investigating the intricate and unstructured brain dynamics involved in design creativity using various neuroimaging tools such as electroencephalography (EEG).

In a nonlinear evolutionary model of design creativity, it is suggested that the brain handles problems and ideas in a way that leads to unpredictable and potentially creative solutions (Zeng, 2001; Nguyen and Zeng, 2012). This involves cognitive processes like thinking of ideas, evolving and evaluating them, along with physical actions like drawing (Zeng et al., 2004; Jia, 2021). This indicates that the brain, as a complex and nonlinear system with characteristics like emergence and self-organization, goes through several cognitive processes which enable the generation of creative ideas and solutions. Exploring brain activities during design creativity tasks helps us get a better insight into the design process and improves how designers perform. As a result, design neurocognition combines traditional design study methods with approaches from cognitive neuroscience, neurophysiology, and artificial intelligence, offering unique perspectives on understanding design thinking (Balters et al., 2023). Although several studies have focused on design and creativity, brain dynamics associated with design creativity are largely untouched. It motivated us to conduct this literature review to explore the studies, gather the information and findings, and finally discuss them. Due to the advantages of electroencephalography (EEG) in design creativity experiments which will be explained in the following paragraphs, we decided to focus on EEG-based neurocognition in design creativity.

As mentioned before, design creativity tasks are cognitive activities which are complex, dynamic, nonlinear, self-organized, and emergent. The brain dynamics of design creativity are largely unknown. Brain behavior recognition during design-oriented tasks helps scientists investigate neural mechanisms, vividly understand design tasks, enhance whole design processes, and better help designers (Nguyen and Zeng, 2014a,b, 2017; Liu et al., 2016; Nguyen et al., 2018, 2019; Zhao et al., 2018, 2020; Jia, 2021; Jia et al., 2021; Jia and Zeng, 2021). Exploring brain neural circuits in design-related processes has recently gained considerable attention in different fields of science. Several studies have been conducted to decode brain activity in different steps of design creativity (Petsche et al., 1997; Nguyen and Zeng, 2010, 2014a,b, 2017; Liu et al., 2016; Nguyen et al., 2018; Vieira et al., 2019). Such attempts will lead to investigating the mechanism and nature of the design creativity process and consequently enhance designers’ performance (Balters et al., 2023). The main question of the studies performed in design creativity neurocognition is whether and how we can explore brain dynamics and infer designers’ cognitive states using neuro-cognitive and physiological data like EEG signals.

Neuroimaging is a vital tool in understanding the brain’s structure and function, offering insights into various neurological and psychological conditions. It employs a range of techniques to visualize the brain’s activity and structure. Neuroimaging methods mainly include magnetic resonance imaging (MRI), computed tomography (CT), electroencephalography (EEG), functional near-infrared spectroscopy (fNIRS), functional MRI (fMRI), and magnetoencephalography (MEG). Neuroimaging techniques have helped researchers explore brain dynamics in complex cognitive tasks, one of which is design creativity (Nguyen and Zeng, 2014b; Gao et al., 2017; Zhao et al., 2020). While several neuroimaging methods exist to study brain activity, electroencephalography (EEG) is one of the best methods which has been widely used in several studies in different applications. EEG, as an inexpensive and simple neuroimaging technique with a high temporal resolution and an acceptable spatial resolution, has been used to infer designers’ cognitive and emotional states. Zangeneh Soroush et al. (2023a,b) have recently introduced two comprehensive datasets encompassing EEG recordings in design and creativity experiments, stemmed from several EEG-based design and design creativity studies (Nguyen and Zeng, 2014a; Nguyen et al., 2018, 2019; Jia, 2021; Jia et al., 2021; Jia and Zeng, 2021). In this paper, we review some of the most fundamental studies which have employed electroencephalography (EEG) to explore brain behavior in creativity and design creativity tasks.

### 1.3 EEG approach to studying creativity neurocognition

EEG stands out as a highly promising method for investigating brain dynamics across various fields, including cognitive, clinical, and computational neuroscience studies. In the context of design creativity, EEG offers a valuable means to explore brain activity, particularly considering the physical movements inherent in the design process. However, EEG analysis poses challenges due to its complexity, nonlinearity, and susceptibility to various artifacts. Therefore, gaining a comprehensive understanding of EEG and mastering its utilization and processing is crucial for conducting effective experiments in design creativity research. This review aims to examine studies that have utilized EEG in investigating design creativity tasks.

EEG is a technique for recording the electrical activity of the brain, primarily generated by neuronal firing within the human brain. This activity is almost always captured non-invasively from the scalp in most cognitive studies, though intracranial EEG (iEEG) is recorded inside the skull, for instance in surgical planning for epilepsy. EEG signals are the result of voltage differences measured across two points on the scalp, reflecting the summed synchronized synaptic activities of large populations of cortical neurons, predominantly from pyramidal cells (Teplan, 2002; Sanei and Chambers, 2013).

While the spatial resolution of EEG is relatively poor, EEG offers excellent temporal resolution, capturing neuronal dynamics within milliseconds, a feature not matched by other neuroimaging modalities like functional Near-Infrared Spectroscopy (fNIRS), Positron Emission Tomography (PET), or functional Magnetic Resonance Imaging (fMRI).

In contrast, fMRI provides much higher spatial resolution, offering detailed images of brain activity by measuring blood flow changes associated with neuronal activity. However, fMRI’s temporal resolution is lower than EEG, as hemodynamic responses are slower than electrical activities. PET, like fMRI, offers high spatial resolution and involves tracking a radioactive tracer injected into the bloodstream to image metabolic processes in the brain. It is particularly useful for observing brain metabolism and neurochemical changes but is invasive and has limited temporal resolution. fNIRS, measuring hemodynamic responses in the brain via near-infrared light, stands between EEG and fMRI in terms of spatial resolution. It is non-invasive and offers better temporal resolution than fMRI but is less sensitive to deep brain structures compared to fMRI and PET. Each of these techniques, with their unique strengths and limitations, provides complementary insights into brain function (Baillet et al., 2001; Sanei and Chambers, 2013; Choi and Kim, 2018; Peng, 2019).

This understanding of EEG, from its historical development by Hans Berger in 1924 to modern digital recording and analysis techniques, underscores its significance in studying brain function and diagnosing neurological conditions. Despite advancements in technology, the fundamental methods of EEG recording have remained largely unchanged, emphasizing its enduring relevance in neuroscience (Teplan, 2002; Choi and Kim, 2018).

### 1.4 Objectives and structure of the paper

Balters et al. (2023) conducted a comprehensive systematic review including 82 papers on design neurocognition covering nine topics and a large variety of methodological approaches in design neurocognition. A systematic review (Pidgeon et al., 2016), reported several EEG-based studies on functional neuroimaging of visual creativity. Although such a comprehensive review exists in the field of design neurocognition, just a few early reviews focused on creativity neurocognition (Fink and Benedek, 2014, 2021; Benedek and Fink, 2019).

The present review not only reports the studies but also critically discusses the previous findings and results. The rest of this paper is organized as follows: Section 2 introduces our review methodology; Section 3 presents the results from our review process, and Section 4 discusses the major implications of the existing design creativity neurocognition research in future studies. Section 5 concludes the paper.

## 2 Methods and materials

**Figure 1:**
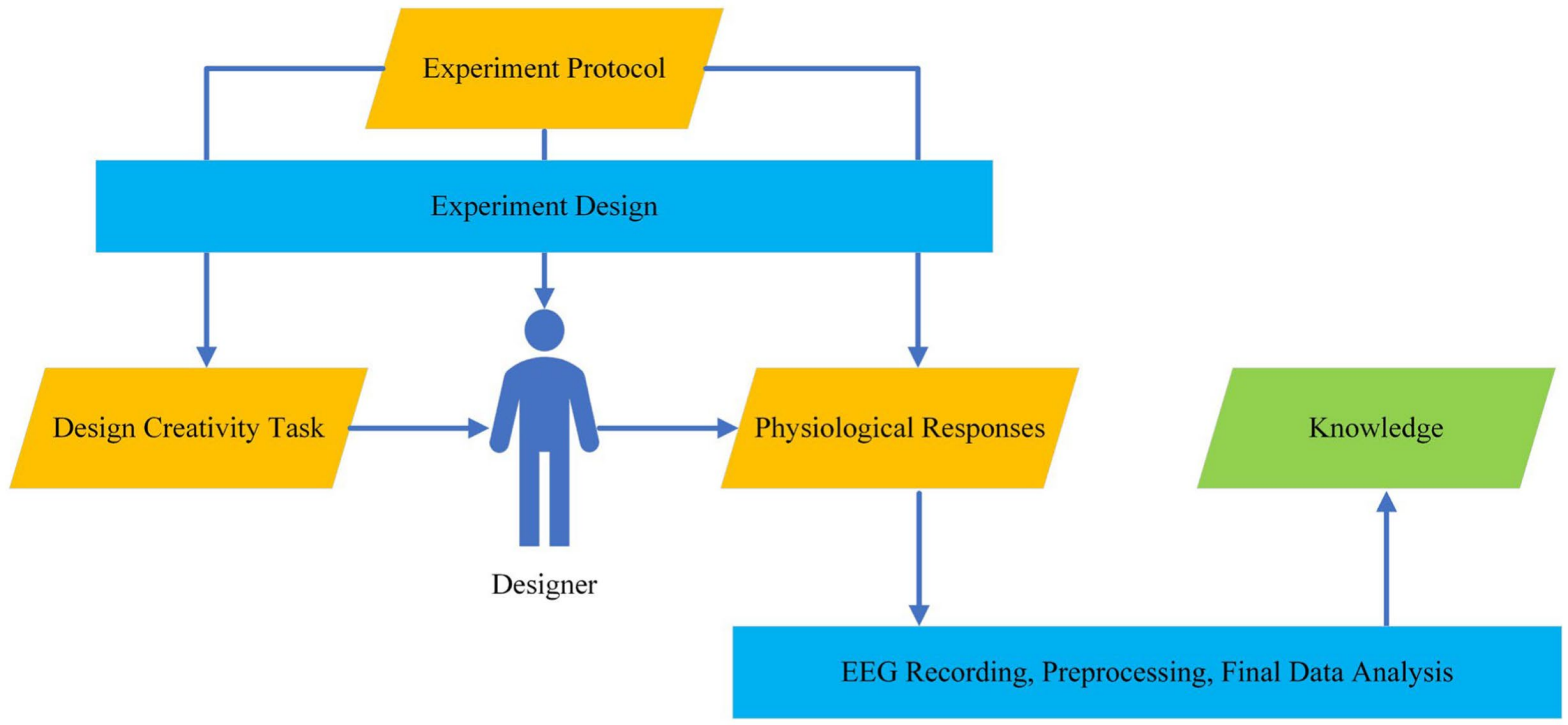
The main components of EEG-based design creativity studies.

Figure 1 shows the main components of EEG-based design creativity studies: (1) experiment design, (2) participants, (3) psychometric tests, (4) experiments (creativity tasks), (5) EEG recording and analysis methods, and (6) final data analysis. The experiment design consists of experiment protocol which includes (design) creativity tasks, the criteria to choose participants, the conditions of the experiment, and recorded physiological responses (which is EEG here). Setting and adjusting these components play a crucial role in successful experiments and reliable results. In this paper, we review studies based on the components in Figure 1.

The components described in Figure 1 are consistent with the stress-effort model proposed by Nguyan and Zeng (Nguyen and Zeng, 2012; Zhao et al., 2018; Yang et al., 2021) which characterizes the relationship between mental stress and mental effort by a bell-shaped curve. This model defines mental stress as a ratio of the perceived task workload over the mental capability constituted by affect, skills, and knowledge. Knowledge is shaped by individual experience and understanding related to the given task workload. Skills encompass thinking styles, strategies, and reasoning ability. The degree of affect in response to a task workload can influence the effective utilization of the skills and knowledge. We thus used this model to form our research questions, determine the keywords, and conduct our analysis and discussions.

### 2.1 Research questions

We focused on the studies assessing brain function in design creativity experiments through EEG analysis. For a comprehensive review, we followed a thorough search strategy, called thematic analysis (Braun and Clarke, 2012), which helped us to code and extract themes from the initial (seed) papers. We began without a fixed topic, immersing ourselves in the existing literature to shape our research questions, keywords, and search queries. Our research questions formed the search keywords and later the search inquiries.

Our main research questions (RQs) were:

RQ1: What are the effective experiment design and protocol to ensure high-quality EEG-based design creativity studies?

RQ2: How can we efficiently record, preprocess, and process EEG reflecting the cognitive workload associated with design creativity tasks?

RQ3: What are the existing methods to analyze the data extracted from EEG signals recorded during design creativity tasks?

RQ4: How can EEG signals provide significant insight into neural circuits and brain dynamics associated with design creativity tasks?

RQ5: What are the significant neuroscientific findings, shortcomings, and inconsistencies in the literature?

With the initial information extracted from the seed papers and the previous studies by the authors in this field (Nguyen and Zeng, 2012, 2014a,b; Jia et al., 2021; Jia and Zeng, 2021; Yang et al., 2022; Zangeneh Soroush et al., 2024), we built a conceptual model represented by Figure 1 and then formed these research questions. With this understanding and the RQs, we set our search strategy.

### 2.2 Search strategy and inclusion-exclusion criteria

Our search started with broad terms like “design,” “creativity,” and “EEG.” These terms encapsulate the overarching cognitive activities and physiological measurement. As we identified relevant papers, we refined our search keywords for a more targeted search. We utilized the Boolean operators such as “OR” and “AND” to finetune our search inquiries. The search inquiries were enhanced by the authors through the knowledge they obtained through selected papers. The first phase started with thematic analysis and continued with choosing papers, obtaining knowledge, discussing the keywords, and updating the search inquiries, recursively until reaching an appropriate search inquiry which resulted in the desired search results. We applied the thematic analysis only in the first iteration to make sure that we had the right and comprehensive understanding of EEG-based design creativity, the appropriate set of keywords, and search inquiries. Finally, we came up with a comprehensive search inquiry as follows:

(“EEG” OR “Electroenceph*” OR “brain” OR “neur*” OR “neural correlates” OR “cognit*”) AND (“design creativity” OR “ideation” OR “creative” OR “divergent thinking” OR “convergent thinking” OR “design neurocognition” OR “creativity” OR “creative design” OR “design thinking” OR “design cognition” OR “creation”)

The search inquiry is a combination of terminologies related to design and creativity, as well as terminologies about EEG, neural activity, and the brain. In a general and quick evaluation, we found out that our proposed search inquiry resulted in relevant studies in the field. This evaluation was a quick way to check how effectively our search keywords work. Then, we went through well-known databases such as PubMed, Scopus, and Web of Science to collect a comprehensive set of original papers, theses, and reviews. These electronic databases were searched to reduce the risk of bias, to get more accurate findings, and to provide coverage of the literature. We continued our search in the aforementioned databases until no more significant papers emerged from those specific databases. It is worth mentioning that we do not consider any specific time interval in our search procedure. We used the fields “title,” “abstract,” and “keywords” in our search process. Then, we selected the papers based on the following inclusion criteria:

1. The paper should answer one or more research questions (RQ1-RQ5).
2. The paper must be a peer-reviewed journal paper authored in English.
3. The paper should focus on EEG analysis related to creativity or design creativity for adult participants.
4. The paper should be related to creativity or design creativity in terms of the concepts, experiments, protocols, and probable models employed in the studies.
5. The paper should use established creativity tasks, including the Alternative Uses Task (AUT), the Torrance Tests of Creative Thinking (TTCT), or a specific design task. (These tasks will be detailed further on.)
6. The paper should include a quantitative analysis of EEG signals in the creativity or design creativity domain.
7. In addition to the above-mentioned criteria, the authors checked the papers to make sure that the included publications have the characteristics of high-quality papers.

These criteria were used to select our initial papers from the large set of papers we collected from Scopus, Web of Science, and PubMed. It should be mentioned that conflicts were resolved through discussion and duplicate papers were removed.

After our initial selection, we used Google Scholar to perform a forward and backward snowball search approach. We chose the snowball search method over the systematic review approach as the forward and backward snowball search methodologies offer efficient alternatives to a systematic review. Unlike systematic reviews, the snowball search method is particularly valuable when dealing with emerging fields or when the scope of inquiry is evolving, allowing researchers to quickly uncover pertinent insights and form connections between seminal and contemporary works. During each iteration of the snowball search, we applied the aforementioned criteria to include or exclude papers accordingly. We continued our snowball search procedure until it converged to the final set of papers. After repeating this over six iterations, we found no new and significant papers, suggesting we had reached a convergent set of papers.

By October 1^st^ (2023), our search was complete. We then organized and studied the final included publications.

## 3 Results

### 3.1 Search results

**Figure 2:**
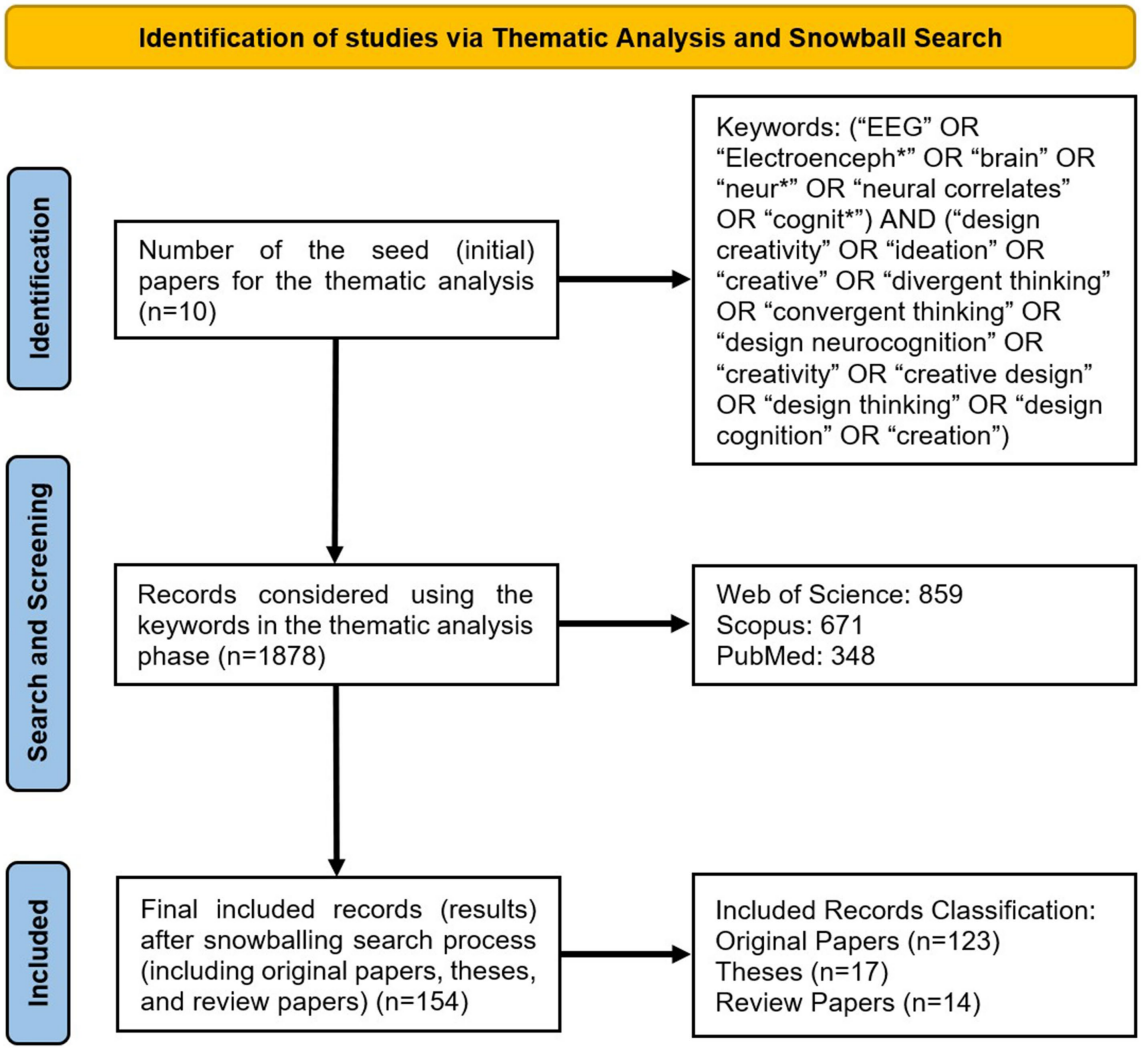
Search procedure and results (adopted from PRISMA) using the thematic analysis in the first iteration and snowball search in the following iterations.

Figure 2 illustrates the general flow of our search procedure, adapted from PRISMA guidelines (Liberati et al., 2009). With the search keywords, we identified 1878 studies during the thematic analysis phase. We considered these studies to select the seed papers for the further snowball search process. After performing the snowball search and considering inclusion and exclusion criteria, we finally selected 154 studies including 82 studies related to creativity (comprising 60 original papers, 12 theses, and 10 review papers) and 72 studies related to design creativity (comprising 63 original papers, 5 theses, and 4 review papers). In our search, we also found 6 related textbooks and 157 studies using other modalities (such as fMRI, fNIRS, etc.) which were excluded. We used these textbooks, theses, and their resources to gain more knowledge in the initial steps of our review. Some studies using fMRI and fNIRS were used to evaluate the results in the discussion. In the snowball search process, a large number of studies have consistently appeared across all iterations implying their high relevance and influence in the field. These papers, which have been repeatedly selected throughout the search process, demonstrate their significant contributions to the understanding of design creativity and EEG studies. The snowball process effectively identifies such pivotal studies by highlighting their recurrent presence and citation in the literature, underscoring their importance in shaping the research landscape.

### 3.2 Design creativity neurocognition: history and trend

As discussed in Section 1, creativity and design creativity studies are different yet closely related in that design creativity involves a more complex design process. In this subsection, we will look at how the design neurocognition creativity study followed the creativity neurocognition study (though not necessarily in a causal manner).

#### 3.2.1 History of creativity neurocognition

Three early studies in the field of creativity neurocognition are Martindale and Mines (1975), Martindale and Hasenfus (1978), and Martindale et al. (1984). In the first study (Martindale and Mines, 1975), it is stated that creative individuals may exhibit certain traits linked to lower cortical activation. This research has shown distinct neural activities when participants engage in two creativity tasks: the Alternate Uses Tasks (AUT) and the Remote Associate Task (RAT). The AUT, which gauges ideational fluency and involves unfocused attention, is related to higher alpha power in the brain. Conversely, the RAT, which centers on producing specific answers, shows varied alpha levels. Previous psychological research aligns with these findings, emphasizing the different nature of these tasks. Creativity, as determined by both tests, is associated with high alpha percentages during the AUT, hinting at an association between creativity and reduced cortical activation during creative tasks. However, highly creative individuals also show a mild deficit in cortical self-control, evident in their increased alpha levels, even when trying to suppress them. This behavior mirrors findings from earlier and later studies and implies that these individuals might have a predisposition to disinhibition. The varying alpha levels during cognitive tasks likely stem from their reaction to tasks rather than intentional focus shifts (Martindale and Mines, 1975).

In the second study (Martindale and Hasenfus, 1978), the authors explored the relationship between creativity and EEG alpha band presence during different stages of the creative process. There were two experiments in this study. Experiment 1 found that highly creative individuals had lower alpha wave presence during the elaboration stage of the creative process, while Experiment 2 found that effort to be original during the inspiration stage was associated with higher alpha wave presence. Overall, the findings suggest that creativity is associated with changes in EEG alpha wave presence during different stages of the creative process. However, the relationship is complex and may depend on factors such as effort to be original and the specific stage of the creative process.

Finally, a series of three studies indicated a link between creativity and hemispheric asymmetry during creative tasks (Martindale et al., 1984). Creative individuals typically exhibited heightened right-hemisphere activity compared to the left during creative output. However, no distinct correlation was found between creativity and varying levels of hemispheric asymmetry during the inspiration versus elaboration phases. The findings suggest that this relationship is consistent across different stages of creative production. These findings were the foundation of design creativity studies which were more explored later and confirmed by other studies (Petsche et al., 1997). Later studies have used these findings to validate their results. In addition to these early studies, there exist several reviews such as Fink and Benedek (2014), Pidgeon et al. (2016), and Rominger et al. (2022a) which provide a comprehensive literature review of previous studies and their main findings including early studies as well as recent creativity research.

#### 3.2.2 EEG-based creativity studies

In the preceding sections, we aimed to lay a foundational understanding of neurocognition in creativity, equipping readers with essential knowledge for the subsequent content. In this subsection, we will briefly introduce the main and most important points regarding creativity experiments. More detailed information can be found in Simonton (2000), Srinivasan (2007), Arden et al. (2010), Fink and Benedek (2014), Pidgeon et al. (2016), Lazar (2018), and Hu and Shepley (2022).

This section presents key details from the selected studies in a structured format to facilitate easy understanding and comparison for readers. As outlined earlier, crucial elements in creativity research include the participants, psychometric tests used, creativity tasks, EEG recording and analysis techniques, and the methods of final data analysis. We have organized these factors, along with the principal findings of each study, into Table 1. This approach allows readers to quickly grasp the essential information and compare various aspects of different studies. The table format not only aids in presenting data clearly and concisely but also helps in highlighting similarities and differences across studies, providing a comprehensive overview of the field. Following the table, we have included a discussion section. This discussion synthesizes the information from the table, offering insights and interpretations of the trends, implications, and significance of these studies in the broader context of creativity neurocognition. This structured presentation of studies, followed by a detailed discussion, is designed to enhance the reader’s understanding, and provide a solid foundation for future research in this dynamic and evolving field.

**Table 1:** A summary of EEG-based creativity neurocognition studies.

Ref	Participants	Psychometric tests	Creativity experiment	EEG/Data analysis	Main findings
Petsche et al. (1997)	38	Wiener test system (for mood) before and after the experiment, Schmidt-Henrich intelligent test	Mental tasks concerning visual perception and imagery; listening to and composing music; verbal and visual creativity; and aspects of mood	FFT and coherence analysis for all possible electrode pairs in all EEG frequency bands	Fewer differences between channel pairs for amplitude than for coherence. Significant differences in lower and upper alpha in all the visual perception tasks.
Jausovec and Jausovec (2000)	Experiment 1 (27) and Experiment 2 (30)	Not Applicable (N/A)	AUT and variant of TTCT	EEG spectral power and coherence, upper and lower alpha band amplitude, and coherence analysis	Significant differences between figural and verbal tasks in EEG power. Coherence was associated with the level of creativity. Noticeable increase in intra- and interhemispheric cooperation between mainly the far distant brain regions while solving the dialectic problems.
Razumnikova et al. (2009)	53	N/A	26 participants performed the figural creativity tasks (TTCT), 27 performed a modified version	FFT, power, and spectral analysis in EEG subbands	Higher upper alpha activity for women in the verbal creativity task and higher beta activity for men in the figural creativity task.
Volf and Tarasova (2010)	28	N/A	TTCT	Baseline power and event-related desynchronization/synchronization (ED/ES) of theta and beta bands	The ED/ES of the upper theta and beta bands of the subjects were dependent on the level of creativity and the person’s sex only in response to the instruction “to create images.” Upper theta in temporal–parietal–occipital brain regions was associated with the originality scores in male participants (unlike female ones). Significant differences in participants with different levels of originality in lower and upper beta. The high level of creativity in men and women is related to sex-dependent specific patterns of frontal–occipital and lateral activities of theta and beta bands.
Volf et al. (2010)	40	N/A	TTCT	FFT, coherence, and spectral analysis of EEG in lower and upper alpha band	Differences in coherence changes during task performance were observed in individuals with varying levels of originality, particularly at theta2, alpha1, and alpha2 frequencies. Notably, lower originality levels correlated with decreased coherence, while higher originality levels showed a less significant decrease in the alpha2 range.
					Gender, creativity, laterality, and electrode position factors interacted in the alpha2 range during task-related intrahemisphere coherence analysis. Spatial distribution patterns were similar between men and women with opposite creativity levels, but high-creativity men exhibited more substantial task-linked coherence changes in specific brain areas compared to those with low creativity.
Mastria et al. (2021)	20	N/A	AUT	EEG lower alpha synchronization	Switching ideas, compared to staying in the same category of ideas, is associated with greater activity in the lower alpha band in the left hemisphere.
Fink and Neubauer (2006)	31	Verbal IQ, Intelligenz-Struktur-Test 2000-R, participants’ personality traits, NEO-FFI, STAI	Two verbal creativity tasks: solving verbal problems and imagining utopian situations	TRP analysis of the EEG alpha band	Creative problem-solving is correlated with an increase in alpha power. More original responses were associated with stronger task-related alpha synchronization in posterior cortices. Creative problem-solving is correlated with sex and IQ.
Wang et al. (2017)	24	N/A	AUT, letter-memory task, the Stroop color-word-interference task	ERD/ERS; upper alpha (10–13 Hz) synchronization, particularly in the left frontal areas of the brain	Individuals with higher shifting abilities produced more original ideas in the later stages of divergent thinking (DT). Individuals with lower inhibition exhibited stronger upper alpha synchronization in the left frontal areas during the early stage compared to the later stage.
Benedek et al. (2011)	30	N/A	Both convergent and divergent thinking tasks in two experimental conditions involving either low or high internal processing demands (2 × 2within-subject design)	EEG alpha synchronization, particularly in the frontal region.	Frontal alpha synchronization occurred during both convergent and divergent thinking, but only under conditions of high internal processing demands. Frontal alpha synchronization is linked more to top-down control.
Benedek et al. (2014)	40	N/A	The four-word sentences (FS) task and AUT	EEG alpha power, particularly in the right parietal cortex	Increases in alpha power in the right parietal cortex are indicative of focused internal attention.
Rominger et al. (2018)	50	N/A	TTCT	TRP for the alpha band, and statistical analysis	Strong desynchronization of upper alpha power during creative ideation. A relative increase in upper alpha power at parietal and occipital sites during idea elaboration.
Camarda et al. (2018)	24	N/A	AUT	Alpha synchronization in both the frontal and temporo-parietal regions	In design fixation, high originality scorers maintained alpha synchronization in temporo-parietal regions, while low scorers displayed alpha desynchronization in these areas. In the control group, participants with high originality scores maintained frontal alpha synchronization, while those with low scores showed a decrease.
Prent and Smit (2020)	40	N/A	AUT	Temporal autocorrelations of the amplitude modulation of the dominant alpha oscillations (8–13 Hz)	Significant negative correlations between creativity and temporal autocorrelations over right central/temporal brain areas.
Mazza et al. (2023)	32	N/A	AUT	Reaction time, EEG alpha, beta, gamma bands analysis, pupil dilation and eye gaze	Participants engaged in divergent thinking took a longer time to generate uncommon uses for everyday objects as compared to convergent thinking. During divergent thinking, participants showed alpha synchronization along with beta and gamma desynchronization, more pronounced leftward gaze shift, and greater pupil dilation. In contrast, convergent thinking displayed desynchronization in alpha and an increase in beta and gamma rhythm, along with a reduction of leftward gaze shift and greater pupil constriction.
Rominger et al. (2022a)	100	N/A	AUT	TRP	During creative ideation and idea evaluation, the TRP was lower at temporal/central and parietal/occipital areas compared to frontal areas. Temporal/central sites showed higher TRP compared to parietal/occipital areas. Participants with higher monitoring skills and creative potential showed stronger alpha power decreases at parietal/occipital sites during creative idea generation and evaluation. Those with lower metacognitive monitoring skills showed alpha power increases.
Rominger et al. (2020)	50	N/A	Figural TTCT	TRP, EEG task-related phase-locking in the upper-alpha range, functional connectivity between brain regions, focusing on frontal-central and frontal-temporal, frontal–parietal/occipital networks	An increase in functional coupling from idea generation to elaboration, most pronounced in frontal-central and frontal-temporal networks. In idea generation, an increase in the connectivity in the frontal–parietal/occipital network and constant connectivity in idea elaboration.
Grabner et al. (2007)	26	N/A	Verbal TTCT	ERS/ERD of lower and upper alpha band, phase locking	More original ideas correlated with increased alpha synchronization and phase coupling in the right hemisphere.
Fink et al. (2011)	45	N/A	AUT	EEG alpha synchronization	Creative cognition generally resulted in alpha synchronization, mainly in the prefrontal cortex and right hemisphere. Both cognitive and affective interventions led to stronger prefrontal alpha activity in the upper alpha band than in the control condition.
Jauk et al. (2012)	55	Intelligenzstrukturanalyse for intelligence, German version of the Eysenck Personality, Stait-Trait-Anxiety-Inventory	AUT, word processing task	TRP, EEG alpha synchronization in the left and right hemisphere	Divergent processing is associated with higher task-related EEG alpha power as compared to convergent processing in both the word association task and AUT.
Gubler et al. (2023)	76	N/A	AUT	TRP, Statistical analysis	No significant difference in AUT performance between the pain and pain-free groups. The pain group showed more pronounced TRP increases in the upper alpha band at the right temporal, parietal, and occipital sites.
Rominger et al. (2022b)	74	N/A	AUT	Task-related alpha power	More creative ideas are associated with increased power in the right posterior region of the brain and enhanced coupling between the frontal, parietal, and occipital regions in the upper alpha band.
Stevens and Zabelina (2020)	29	N/A	AUT	Temporal and spectral analysis	More creative individuals and more creative task conditions are associated with greater EEG alpha power.
Perchtold-Stefan et al. (2020)	93	Humor Comprehension Task (HUT)	AUT	Task-related changes in EEG alpha power, brain topography, statistical analysis	An increase in task-related alpha power in HUT and AUT, more right-lateralized at ventral fronto-temporal sites in the HUT than AUT.
Hao et al. (2016)	20	The Chinese version of the Spielberger’s state–trait anxiety inventory (STAI)	AUT	ERD/ERS	Idea evaluation is associated with upper alpha synchronization at the frontal cortical regions. Upper alpha activity in frontal cortices during idea generation was enhanced after idea evaluation.
Agnoli et al. (2020)	20	N/A	AUT	Alpha TRP	A shift from alpha desynchronization to synchronization during idea creation. Alpha power changes linked to response originality, varying by brain region. Early DT phases showed alpha synchronization in frontal, central, and temporal areas indicating original ideas. Centro-parietal alpha synchronization consistently predicted originality throughout DT. Bilateral frontal and left-sided central, temporal, and parietal effects were key to increasing response originality.
Rominger et al. (2019)	86	Structured Clinical Interview for DSM-IV Axis I Disorders (SCID screening)	AUT	EEG phase locking, TRP, statistical analysis	The U-shaped alpha power trend is associated with an increase in functional communication between frontal and parietal-occipital brain regions.
Schwab et al. (2014)	45	N/A	AUT	Upper alpha band: task-related power (TRP)	A general increase in alpha power at the beginning of idea generation followed by a decrease, and then a re-increase just before responding, particularly noticeable at the right hemisphere’s parietal and temporal sites. The production of more original ideas correlated with increasing hemispheric asymmetry, showing more alpha activity in the right hemisphere compared to the left, especially as the idea generation period progressed.
Jia and Zeng (2021)	29	N/A	Modified figural TTCT	Microstate analysis, TRP	A general decrease in alpha power across all thinking modes compared to rest. The lower alpha band (8–10 Hz) showed significantly less decrease in power during idea evolution. The upper alpha band (10–12 Hz) showed more decrease in power over central sites during evaluation, indicating higher task-specific demands.
Yin et al. (2023)	30	N/A	AUT	ERP analysis, statistical analysis, brain topography maps	The study introduces the correlations between cognitive tasks (retrieval, recall, combination, and association, combination) and creative design events. The decoding method reliably reports recall and association occurrences. Association emerges as the primary cognitive factor for superior creative output quality. Recall predominates for lower levels of creative output quality.
Ahad et al. (2023)	10	N/A	Modified AUT	Event-related potentials (ERPs), TRP, EEG alpha power, statistical analysis (ANOVA), machine learning (k-nearest neighbor)	~99.9% of classification performance in the classification of participants’ neural responses Larger N400 amplitudes for nonsensical and creative stimuli compared to common uses within the 300–500 ms window. ANOVA analysis indicated a significant main effect: decreased alpha power during creative ideation, particularly over the parietooccipital temporal area (O1/2, P7/8).

In our research, we initially conducted a thematic analysis and utilized a forward and backward snowball search method to select relevant studies. Out of these, five studies employed machine learning techniques, while the remaining ones concentrated on statistically analyzing EEG features. It is noteworthy that all the chosen studies followed a similar methodology, involving the recruitment of participants, administering probable psychometric tests, conducting creativity tasks, recording EEG data, and concluding with final data analysis.

While most studies follow similar structure for their experiments, some other studies focus on other aspects of creativity such as artistic creativity and poetry, targeting different evaluation methods, and through different approaches. In Shemyakina and Dan’ko (2004) and Danko et al. (2009), the authors targeted creativity to produce proverbs or definitions of emotions of notions. In other studies (Leikin, 2013; Hetzroni et al., 2019), the experiments are focused on creativity and problem-solving in autism and bilingualism. Moreover, some studies such as Volf and Razumnikova (1999) and Razumnikova (2004) focus more on the gender differences in brain organization during creativity tasks. In another study (Petsche, 1996), approaches to verbal, visual, and musical creativity were explored through EEG coherence analysis. Similarly, the study (Bhattacharya and Petsche, 2005) analyzed brain dynamics in mentally composing drawings through differences in cortical integration patterns between artists and non-artists. We summarized the findings of EEG-based creativity studies in Table 1.

#### 3.2.3 Neurocognitive studies of design and design creativity

Design is closely associated with creativity. On the one hand, by definition, creativity is a measure of the process of creating, for which design, either intentional or unconscious, is an indispensable constituent. On the other hand, it is important to note that not all designs are inherently creative; many designs follow established patterns and resemble existing ones, differing only in their specific context. Early research on design creativity aimed to differentiate between design and design creativity tasks by examining when and how designers exhibited creativity in their work. In recent years, much of the focus in design creativity research has shifted towards cognitive and neurocognitive investigations, as well as the development of computational models to simulate creative processes (Borgianni and Maccioni, 2020; Lloyd-Cox et al., 2022). Neurocognitive studies employ neuroimaging methods (such as EEG) while computational models often leverage artificial intelligence or cognitive modeling techniques (Zeng and Yao, 2009; Gero, 2020; Gero and Milovanovic, 2020). In this section, we review significant EEG-based studies in design creativity to focus more on design creation and highlight the differences. While most studies have processed EEG to provide more detailed insight into brain dynamics, some studies such as Goel (2014) outlined a preliminary framework encompassing cognitive and neuropsychological systems essential for explaining creativity in designing artifacts.

Several studies have recorded and analyzed EEG in design and design creativity tasks. Most neuro-cognitive studies have directly or indirectly employed frequency-based analysis which is based on the analysis of EEG in specific frequency bands including delta (0.5–4 Hz), theta (4–8 Hz), alpha (8–13 Hz), beta (13–30 Hz), and gamma (>30 Hz). One of the main analyses is called task-related potential (TRP) which has provided a foundation for other analyses. It computes the relative power of the EEG signal associated with a design task in a specific frequency band with respect to the power of EEG in the rest mode. This analysis is simple and effective and reveals the physiological processes underlying EEG dynamics (Rominger et al., 2018; Jia and Zeng, 2021; Gubler et al., 2022; Rominger et al., 2022b).

Frequency-based analyses have been widely employed. For example, the study (Borgianni and Maccioni, 2020) applied TRP analysis to compare the neurophysiological activations of mechanical engineers and industrial designers while conducting design tasks including problem-solving, basic design, and open design. These studies have agreed that higher alpha band activity is sensitive to specific task-related requirements, while the lower alpha corresponds to attention processes such as vigilance and alertness (Klimesch et al., 1998; Klimesch, 1999; Chrysikou and Gero, 2020). Higher alpha activity in the prefrontal region reflects complex cognitive processes, higher internal attention (such as in imagination), and task-irrelevant inhibition (Fink et al., 2009a,b; Fink and Benedek, 2014). On the other hand, higher alpha activity in the occipital and temporal lobes corresponds to visualization processes (Vieira et al., 2022a). In design research, to compare EEG characteristics in design activities (such as idea generation or evaluation) (Liu et al., 2016), frequency-based analysis has been widely employed (Liu et al., 2018). Higher alpha is associated with open-ended tasks, visual association in expert designers, and divergent thinking (Nguyen and Zeng, 2014b; Nguyen et al., 2019). Higher beta and theta play a pivotal role in convergent thinking, and constraint tasks (Nguyen and Zeng, 2010; Liu et al., 2016; Liang and Liu, 2019).

The research in design and design creativity is not limited to frequency-based analyses. Nguyen et al. (2019) introduced Microstate analysis to EEG-based design studies. Using the microstate analysis, Jia and Zeng investigated EEG characteristics in design creativity experiment (Jia and Zeng, 2021), where EEG signals were recorded while participants conducted design creativity experiments which were modified TTCT tasks (Nguyen and Zeng, 2014b).

Following the same approach, Jia et al. (2021) analyzed EEG microstates to decode brain dynamics in design cognitive states including problem understanding, idea generation, rating idea generation, idea evaluation, and rating idea evaluation, where six design problems including designing a birthday cake, a toothbrush, a recycle bin, a drinking fountain, a workplace, and a wheelchair were used for the EEG based design experimental studies (Nguyen and Zeng, 2017). The data of these two loosely controlled EEG-based design experiments are summarized and available for the research community (Zangeneh Soroush et al., 2024).

We summarized the findings of EEG-based design and design creativity studies in Table 2.

**Table 2:** A summary of EEG-based design creativity neurocognition studies.

Ref	Participants	Psychometric tests	Experiment	EEG/data analysis	Main findings
Kruk et al. (2014)	14	N/A	Two different tasks: clay sculpting and drawing	qEEG frequency-based analysis of the bilateral medial frontal cortex and bilateral me-dial parietal cortex	Both activities increased gamma power in the right medial parietal lobe. Unlike drawing, clay sculpting decreased right medial frontal gamma power and elevated theta power.
Nguyen and Zeng (2014a)	11	N/A	Three design problems including designing (1) a house that can fly, (2) a vehicle that can transport an object between any two locations (3) a desk for a messy university student	14 EEG channels were processed through segmentation and power spectral density	Mental effort is the lowest at a high stress level and there is no significant difference in mental effort between medium stress level and low stress level.
Vieira et al. (2019)	36	N/A	Three tasks including problem-solving, basic design, open design of a set of furniture	TRP	Significant design cognition differences between the mechanical engineering students and architects in task-related power between the problem-solving task and the design tasks.
Liu et al. (2014)	15 and 24 participants in the first and second pilot studies respectively	N/A	A set of computer-aided design (CAD) tasks including a modeling task in Solid Edge™, and configuration and optimization in Siemens NX™	A fuzzy logical model was used to map EEG into four emotions	The fuzzy model was successful in emotion recognition during design tasks
Nguyen et al. (2019)	A subset of eight datasets recorded on eight different subjects	N/A	Six design problems including design: (1) a birthday cake, (2) a wheelchair, (3) a water fountain, (4) a workplace, (5) a recycle bin, and (6) a toothbrush	EEG segmentation using frequency measures and micro state analysis (power spectral density)	An effective and fully automated method for EEG segmentation in design creativity experiments which can complement complex domain expert segmentation. Subjective relationships identified between fuzzy inference system objective and questionnaire/interview subjective emotional measures.
Vieira et al. (2020b)	32 participants including 15 mechanical engineers and 17 industrial designers	N/A	Professional mechanical engineers and industrial designers in two prototypical design tasks, a problem-solving constrained layout task and an open design sketching task	Statistical analysis (ANOVA) of the power values of EEG frequency bands	Significant differences in EEG bands’ activity in stages of the design spaces across and between two groups of participants
Vieira et al. (2022a)	84	N/A	Constrained and open design tasks to design a set of furniture and an open design task including free-hand sketching	Frequency-based analysis in all EEG bands and statistical analysis (ANOVA)	A main effect of sex for theta, alpha 2, and beta 1 frequency bands. Higher theta, alpha 2, and beta 1, for women in both design tasks in the right dorsolateral prefrontal cortex, right occipitotemporal cortex, secondary visual cortex, and prefrontal cortex. Higher beta, in the left prefrontal cortex, for women in the constrained design.
					Women had higher theta, alpha, and beta 2 in the left prefrontal cortex and secondary visual cortex for all frequency bands in the open design. Results within gender between tasks indicate higher theta and alpha in the prefrontal cortex in the constrained design for both genders. Higher theta and alpha 1 in the right hemisphere and higher alpha 2 and beta bands across hemispheres in the open design for both sexes. Common brain areas and frequency bands in distinguishing constrained from open design.
Jia et al. (2021)	27	N/A	Six open-ended loosely controlled design problems including design: (1) a birthday cake, (2) a wheelchair, (3) a water fountain, (4) a workplace, (5) a recycle bin, and (6) a toothbrush	TRP and microstate analysis followed by statistical analysis (ANOVA)	Significant differences in the design tasks in EEG characteristics in cognitive states including problem understanding, idea generation, idea evaluation, rating idea generation, and rating idea evaluation (in both TRP and microstate analysis).
Vieira et al. (2021)	24	N/A	Two tasks including a well-defined problem-solving task with a unique set of solutions and an ill-defined design task	EEG frequency power and statistical analysis	No main effect of sex and a consistent main effect of hemisphere for the six frequency bands. Male designers showed higher alpha and beta in the prefrontal cortices for men and in the right occipitotemporal cortex and secondary visual cortices for women designers. Men show higher alpha and beta in the right prefrontal area in the design sketching stage and women in the right temporal cortex and left prefrontal cortex and higher theta, in the design sketching stage.
Jia and Zeng (2021)	28	N/A	Three open-ended loosely controlled design creation tasks including three steps of idea generation, idea evolution, and idea evaluation	EEG frequency-based analysis, microstate analysis, and statistical analysis (ANOVA)	Cognitive states showed different EEG dynamics in microstate analysis through statistical analysis more effectively compared to frequency-based methods.
Vieira et al. (2020a)	26 mechanical engineers and 29 industrial designers	N/A	Four tasks including problem-solving, basic layout design, open layout design for a set of furniture, open free-hand sketching design	Temporal analysis, TRP, and ANOVA	Significant differences in activations between the problem-solving and open design tasks. Significant differences between open design sketching and the problem-solving. Significant differences between the open sketching design and the problem-solving for industrial designers.
Vieira et al. (2020d)	18 mechanical engineers and 18 industrial designers	N/A	Four tasks including problem-solving, basic layout design, open layout design for a set of furniture, open free-hand sketching design	Frequency-based analysis, EEG power, and statistical analysis (ANOVA)	Significant differences in EEG characteristics between problem-solving and open design sketching in both novice and experienced designers and for both mechanical engineers and industrial designers. Neurophysiological activations of experienced and novice professional designers when problem-solving and designing are significantly different. Experienced professionals show higher transformed power compared to novice designers.
Göker (1997)	8	N/A	Five tasks including some calculation tasks, a range of The Incredible Machine (TIM) design assignments of varying difficulty, decoding a solution to a similar assignment, recognizing some objects, classification of objects	Coherence analysis, brain topography maps, and the measure of relative duration of high interregional coherences (RDHlrCs)	Novices show a longer activity in the frontal regions whereas the experts seem to have longer activity in the parietal regions of the brain. RDHlrCs between the frontal and parietal regions are longer for the expert compared to the novice.
Hu et al. (2022)	12	N/A	A sequence of seven design activities including scenario establishment, scenario shift, problem defining, analogy and inference, synthesis, mutation, and reflection	Frequency-based analysis, TRP, and ANOVA for different brain areas and different EEG bands	During the task of analogy and inference, the parieto-occipital area (with the smallest TRP value) had the most notable changes. Power decrease in the alpha band (ERD) compared to the resting state in problem defining, analogy and inference, synthesis, and reflection and search for solutions. During the synthesis task, the hemisphere had a significant effect on beta band activity (a considerable decrease in beta band power in the right central-temporal area during the synthesis task). Alpha band power had event-related synchronization (ERS) in the scenario task and divergent thinking occupies a dominant position. During the reflection task, the brain area had a significant impact on delta and gamma bands, and the fluctuation in both bands displayed event-related desynchronization (ERD). Power decline in delta and gamma bands during reflection task. During the analogy and inference task, the brain area had significant impacts on theta band. The parietooccipital area had the most obvious and significant changes/differences compared with other areas.
Hu et al. (2017)	42	N/A	One design task: “Generate as many concepts as possible for a device that will aid a student athlete with a leg injury. The athlete needs to be able to do normal campus activities such as go to class, get food, or use the restroom.”	EEG frequency measures, and Support Vector Machines (SVM), Statistical analysis	Predict design outcomes (an accuracy of 70%) and the relationship between EEG data and concept-level measures of novelty, quality, and elaboration. Passive attention, active attention, and mental manipulation are significant predictors of ideation metrics. Distraction is positively correlated with design novelty and may have indirect negative effects on design quality. Highly active attention is correlated with good design quality.
Lukačević et al. (2023)	20	N/A	Visuospatially-intensive design tasks of CAD modelling; when technical systems are presented with orthographic and isometric projections in technical drawings	TRP analysis in alpha, beta, and theta bands, and statistical analysis	Sensitivity of engineers’ brain activity to the visual representation of a technical system interpretation. Significant differences in beta, alpha, and theta (TRP) in interpreting the technical drawings and CAD modeling from them. Theta TRP in the frontal lobe in the right hemisphere is essential in the neurocognitive responses to the orthographic and isometric projections.
Giannopulu et al. (2022)	30	N/A	Mental visualization of constructing a setting using either familiar or abstract physical or virtual objects in real and augmented reality environments and effectively creating a scene in augmented reality	Frequency-based analysis, brain connectivity investigation, statistical analysis	Comparable cortico-cortical neural patterns across real and augmented environments. Synchronous beta and gamma oscillatory activities were observed between frontal and posterior brain regions bilaterally. The results indicated a transient synchronized neural architecture. Design creativity tasks involve interconnected networks rather than being localized in a single brain area.
Eymann et al. (2022)	17	N/A	Assessing shared underlying mechanisms for creativity and fluid intelligence by the creative reasoning task (CRT) and Raven’s Advanced Progressive Matrices (APM)	Time-frequency analysis for the EEG subbands, Statistical analysis for both neurophysiological recordings (EEG) and behavioral recordings in Creative reasoning task (CRT), The working memory version of CRT (CRT-WM), and Raven’s Advanced Progressive Matrices (APM)	Higher fronto-parietal alpha synchronization during divergent compared to convergent thinking, particularly towards the conclusion of the thinking phase. Creativity and fluid intelligence share common underlying mechanisms, independent of working memory processes required by specific task demands.
Kuznetsov et al. (2023)	49	N/A	Performing a mental design creativity divergent thinking task	EEG power spectrum analysis, and source localization through sLORETA	The higher alpha has a significant correlation with the level of creativity. The level of originality is correlated with the activity of lateral-frontoparietal network (L-FPN) structures. In contrast, the default-mode networks (DMN) activity does not differ significantly between the two groups with lower and higher levels of creativity.
Wang et al. (2023)	72	N/A	Designing a ship in virtual reality (VR)	Frequency-based analysis in EEG sub-bands, ANOVA	VR significantly affects immersion, especially with regard to attention. VR was significantly correlated with theta, beta, and gamma brain wave activity. In the VR scenario, increased attention-related and meditation-related brain wave activity and desynchronized alpha waves were recognized. VR had a slight positive effect on attention levels with regard to immersion. VR did not affect operation ability. VR had a small positive effect on the feasibility of the creative process. VR had no significant effect on creative outcomes.
Nguyen et al. (2018)	A subset of eight datasets recorded on eight different subjects	N/A	Six design problems including design: (1) a birthday cake, (2) a wheelchair, (3) a water fountain, (4) a workplace, (5) a recycle bin, and (6) a toothbrush	Microstate analysis, Frequency-based analysis	The study introduced a novel method to quantify effort, fatigue, and concentration during the conceptual design process. The study introduces and discusses four hypotheses; H1: Effort and fatigue are subject to ice-breaking and end of task phenomena, H2: Fatigue and effort follow a capacity model, H3: Fatigue is multidimensional, H4: Concentration follows a modal shift model
Şekerci et al. (2024)	32	N/A	Three stages of designing a house (Residential bathroom design, Residential-bedroom design, and Residential-living room design) including manual, digital, and 2 dimensional designs using CAD	Brain frontal asymmetry and hemisphere activities in the alpha and beta bands for arousal and valence, statistical analysis	The paper introduces a method to recognize interior architecture students’ emotions while using design tools

#### 3.2.4 Trend analysis

The selected studies span a broad range of years, stretching from 1975 (Martindale and Mines, 1975) to the present day, reflecting advancements in neuro-imaging techniques and machine learning methods that have significantly aided researchers in their investigations. From the earliest studies to more recent ones, the primary focus has centered on EEG sub-bands, brain asymmetry, coherence analysis, and brain topography. Recently, machine learning methods have been employed to classify EEG samples into designers’ cognitive states. These studies can be roughly classified into the following distinct categories based on their proposed experiments and EEG analysis methods (Pidgeon et al., 2016; Jia, 2021): (1) visual creativity versus baseline rest/fixation, (2) visual creativity versus non-rest control task(s), (3) individuals of high versus low creativity, (4) generation of original versus standard visual images, (5) creativity in virtual reality vs. real environment, (6) loosely controlled vs. strictly controlled creativity experiments.

The included studies exhibited considerable variation in the tasks utilized and the primary contrasts examined. Some studies employed frequency-based or EEG power analysis to compare brain activity during visual creativity tasks with tasks involving verbal creativity or both verbal and visual tasks. These tasks often entail memory tasks or tasks focused on convergent thinking. Several studies, however, adopted a simpler approach by comparing electrophysiological activity during visual creativity tasks against a baseline fixation or rest condition. Some studies compared neural activities between individuals characterized by high and low levels of creativity, while others compared the generation of original creative images with that of standard creative images. Several studies analyze brain behavior concerning creativity factors such as fluency, originality, and others. These studies typically employ statistical analysis techniques to illustrate and elucidate differences between various creativity factors and their corresponding brain behaviors. This variability underscores the diverse approaches taken by researchers to examine the neural correlates of design creativity (Pidgeon et al., 2016). However, few studies significantly and deeply delved into areas such as gender differences in creativity, creativity among individuals with mental or physical disorders, or creativity in diverse job positions or skill sets. This suggests that there is significant untapped potential within the EEG-based design creativity research area.

In recent years, advancements in fMRI imaging and its applications have led several studies to replace EEG with fMRI to investigate brain behavior. fMRI extracts metabolism, resulting in relatively high spatial resolution compared to EEG. However, it is important to note that fMRI has lower temporal resolution compared to EEG. Despite this difference, the shift towards fMRI highlights the ongoing evolution and exploration of neuroimaging techniques in understanding the neural correlates of design creativity. fMRI studies provide a deep understanding of neural circuits associated with creativity and can be used to evaluate EEG-based studies (Abraham et al., 2018; Japardi et al., 2018; Zhuang et al., 2021).

The emergence of virtual reality (VR) has had a significant impact on design creativity studies, offering a wide range of experimentation possibilities. VR enables researchers to create diverse scenarios and creativity tasks, providing a dynamic and immersive environment for participants (Agnoli et al., 2021; Chang et al., 2022). Through VR technology, various design creativity experiments can be conducted, allowing for novel approaches and innovative methodologies to explore the creative process. This advancement opens up new avenues for researchers to investigate the complexities of design creativity more interactively and engagingly.

Regardless of the significant progress over the past few decades, design and design creativity neurocognitive research is still in its early stages, due to the challenges identified (Zhao et al., 2020; Jia et al., 2021), which is summarized below:

1. Design tasks are open-ended, meaning there is no single correct outcome and countless acceptable solutions are possible. There are no predetermined or optimal design solutions; multiple feasible solutions may exist for an open-ended design task.
2. Design tasks are ill-defined as finding a solution might change or redefine the original task, leading to new tasks emerging.
3. Various emergent design tasks trigger design knowledge and solutions, which in turn can change or redefine tasks further.
4. The process of completing a design task depends on emerging tasks and the perceived priorities for completion.
5. The criteria to evaluate a design solution are set by the solution itself.

While a lot of lessons learned from creativity neurocognitive research can be borrowed to study design and design creativity neurocognition, new paradigms should be proposed, tested, and validated to advance this new discipline. This advancement will in turn move forward creativity neurocognition research.

### 3.3 Experiment protocol

Concerning the model described in Figure 1, we arranged the following sections to cover all the main components of EEG-based design creativity studies. To bring a general picture of the EEG-based design creativity studies, we briefly explain the procedure of such experiments. Since most design creativity neurocognition research inherited more or less procedures in general creativity research, we will focus on AUT and TTCT tasks. The introduction of a loosely controlled paradigm, tEEG, can be found in Zhao et al. (2020), Jia et al. (2021), and Jia and Zeng (2021). Taking a look at Tables 1, 2, it can be inferred that almost all included studies record EEG signals while selected participants are performing creativity tasks. The first step is determining the sample size, recruiting participants, and psychometrics according to which participants get selected. In some of these studies, participants take psychometric tests before performing the creativity tasks for screening or categorization. In this review, the tasks used to gauge creativity are the Alternative Uses Test (AUT) and the Torrance Test of Creative Thinking (TTCT). During these tasks, EEG is recorded and then preprocessed to remove any probable artifacts. These artifact-free EEGs are then processed to extract specific features, which are subsequently subjected to either statistical analysis or machine learning methods. Statistical analysis typically compares brain dynamics across different creativity tasks like idea generation, idea evolution, and idea evaluation. Machine learning, on the other hand, categorizes EEG signals based on associated creativity tasks. The final stage involves data analysis, which aims to deduce how brain dynamics correlate with the creativity tasks given to participants. This data analysis also compares EEG results with psychometric test findings to discern any significant differences in EEG dynamics and neural activity between groups.

#### 3.3.1 Participants

The first factor of the studies is their participants. In most studies, participants are right-handed, non-medicated, and have normal or corrected to normal vision. In some cases, the Edinburgh Handedness Inventory (Oldfield, 1971) (with 11 elements) or hand dominance test (HDT) (Steingrüber et al., 1971) were employed to determine participants’ handedness (Rominger et al., 2020; Gubler et al., 2023; Mazza et al., 2023). While in several creativity studies, right-handedness has been considered; relatively, in design creativity studies it has been less mentioned.

In most studies, participants are undergraduate or graduate students with different educational backgrounds and an age range of 18 to 30 years. In the included papers, participants did not report any history of psychiatric or neurological disorders, or treatment. It should be noted that some studies such as Ayoobi et al. (2022) and Gubler et al. (2022) analyzed creativity in health conditions like multiple sclerosis or participants with chronic pain, respectively. These studies usually conduct statistical analysis to investigate the results of creativity tasks such as AUT or Remote Association Task (RAT) and then associate the results with the health condition. In some studies, it is reported that participants were asked not to smoke cigarettes for 1 h, not to have coffee for 2 h, alcohol for 12 h, or other stimulating beverages for several hours before experiments. As mentioned in some design creativity studies, similar rules apply to design creativity experiments (participants are not allowed to have stimulating beverages).

In most studies, the sample size of participants was as large as 15 up to 45 participants except for a few studies (Jauk et al., 2012; Perchtold-Stefan et al., 2020; Rominger et al., 2022a,b) which had larger numbers such as 100, 55, 93, and 74 participants, respectively. Some studies such as Agnoli et al. (2020) and Rominger et al. (2020) calculated their required sample size through G*power software (Faul et al., 2007) concerning their desirable chance (or power) of detecting a specific interaction effect involving the response, hemisphere, and position (Agnoli et al., 2020). Considering design creativity studies, the same trend can be seen as the minimum and maximum numbers of participants are 8 and 84, respectively. Similarly, in a few studies, sample sizes were estimated through statistical methods such as G*power (Giannopulu et al., 2022).

In most studies, a considerable number of participants were excluded due to several reasons such as not being fluent in the language used in the experiment, left-handedness, poor quality of recorded signals, extensive EEG artifacts, misunderstanding the procedure of the experiment correctly, technical errors, losing the data during the experiment, no variance in the ratings, and insufficient behavioral data. This shows that recording a high-quality dataset is quite challenging as several factors determine whether the quality is acceptable. Two datasets (in design and creativity) with public access have recently been published in Mendeley Data (Zangeneh Soroush et al., 2023a,b). Except for these two datasets, to the best of our knowledge, there is no publicly available dataset of EEG signals recorded in design and design creativity experiments.

Regarding the gender analysis, among the included papers, there were a few studies which directly focused on the association between gender, design creativity, and brain dynamics (Vieira et al., 2021, 2022a). In addition, most of the included papers did not choose the participants’ gender to include or exclude them. In some cases, participants’ genders were not reported.

#### 3.3.2 Psychometric tests

Before the EEG recording sessions, participants are often screened using psychometric tests, which are usually employed to categorize participants based on different aspects of intellectual abilities, ideational fluency, and cognitive development. These tests provide scores on various cognitive abilities. Additionally, personality tests are used to create personas for participants. Self-report questionnaires measure traits such as anxiety, mood, and depression. Some of the psychometric tests include the Intelligenz-Struktur-Test 2000-R (I-S-T 2000 R), which assesses general mental ability and specific intellectual abilities like visuospatial, numerical, and verbal abilities. The big five test is used for measuring personality traits like conscientiousness, extraversion, neuroticism, openness to experience, and agreeableness. Other tests such as Spielberger’s state–trait anxiety inventory (STAI) are used for mood and anxiety, while the Eysenck Personality Questionnaire (EPQ-R) investigates possible personality correlates of task performance (Fink and Neubauer, 2006, 2008; Fink et al., 2009a; Jauk et al., 2012; Wang et al., 2019). To the best of our knowledge, the included design creativity studies have not directly utilized psychometrics (Table 2) to explore the association between participants’ cognitive characteristics and brain behavior. There exist a few studies which have indirectly used cognitive characteristics. For instance, Eymann et al. (2022) assessed the shared mechanisms of creativity and intelligence in creative reasoning and their correlations with EEG characteristics.

#### 3.3.3 Creativity and design creativity tasks

In this section, we introduce some experimental creativity tasks such as the Alternate Uses Task (AUT), and the Torrance Test of Creative Thinking (TTCT). Here, we would like to shed light on these tasks and their correlation with design creativity. One of the main characteristics of design creativity is divergent thinking as its first phase which is addressed by these two creativity tasks. In addition, AUT and TTCT are adopted and modified by several studies such as Hartog et al. (2020), Hartog (2021), Jia et al. (2021), Jia and Zeng (2021), and Li et al. (2021) for design creativity neurocognition studies. The figural version of TTCT is aligned with the goals of design creativity tasks where designers (specifically in engineering domains) create or draw their ideas, generate solutions, and evaluate and evolve generated solutions (Srinivasan, 2007; Mayseless et al., 2014; Jia et al., 2021).

Furthermore, design creativity studies have introduced different types of design tasks from sequence of simple design problems to constrained and open design tasks (Nguyen et al., 2018; Vieira et al., 2022a). This variety of tasks opens new perspectives to the design creativity neurocognition studies. For example, the six design problems have been employed in some studies (Nguyen and Zeng, 2014b); ill-defined design tasks are used to explore brain dynamics differences between novice and expert designers (Vieira et al., 2020d).

The Alternate Uses Task (AUT), established by Guilford (1967), is a prominent tool in psychological evaluations for assessing divergent thinking, an essential element of creativity. In AUT (Guilford, 1967), participants are prompted to think of new and unconventional uses for everyday objects. Each object is usually shown twice – initially in the normal (common) condition and subsequently in the uncommon condition. In the common condition, participants are asked to consider regular, everyday uses for the objects. Conversely, in uncommon conditions, they are encouraged to come up with unique, inventive uses for the objects (Stevens and Zabelina, 2020). The test includes several items for consideration, e.g., brick, foil, hanger, helmet, key, magnet, pencil, and pipe. In the uncommon condition, participants are asked to come up with as many uses as they can for everyday objects, such as shoes. It requires them to think beyond the typical uses (e.g., foot protection) and envision novel uses (e.g., a plant pot or ashtray). The responses in this classic task do not distinguish between the two key elements of creativity: originality (being novel and unique) and appropriateness (being relevant and meaningful) (Runco and Mraz, 1992; Wang et al., 2017). For instance, when using a newspaper in the AUT, responses can vary from common uses like reading or wrapping to more inventive ones like creating a temporary umbrella. The AUT requires participants to generate multiple uses for everyday objects thereby measuring creativity through four main criteria: fluency (quantity of ideas), originality (uniqueness of ideas), flexibility (diversity of idea categories), and elaboration (detail in ideas) (Cropley, 2000; Runco and Acar, 2012). In addition to the original indices of AUT, there are some creativity tests which include other indices such as fluency-valid and usefulness. Usefulness refers to how functional the ideas are (Cropley, 2000; Runco and Acar, 2012) whereas fluency-valid, which only counts unique and non-repeated ideas, is defined as a valid number of ideas (Prent and Smit, 2020). The AUT’s straightforward design and versatility make it a favored method for gauging creative capacity in diverse groups and settings, reflecting its universal applicability in creativity assessment (Runco and Acar, 2012).

Developed by E. Paul Torrance in the late 1960s, the Torrance Test of Creative Thinking (TTCT) (Torrance, 1966) is a foundational instrument for evaluating creative thinking. TTCT is recognized as a highly popular and extensively utilized tool for assessing creativity. Unlike the AUT, the TTCT is more structured and exists in two versions: verbal and figural. The verbal part of the TTCT, known as TTCT-Verbal, includes several subtests (Almeida et al., 2008): (a) Asking Questions and Making Guesses (subtests 1, 2, and 3), where participants are required to pose questions and hypothesize about potential causes and effects; (b) Improvement of a Product (subtest 4), which involves suggesting modifications to the product; (c) Unusual Uses (subtest 5), where participants think of creative and atypical uses; and (d) Supposing (subtest 6), where participants imagine the outcomes of an unlikely event, as per Torrance. The figural component, TTCT-Figural, contains three tasks (Almeida et al., 2008): (a) creating a drawing; (b) completing an unfinished drawing; and (c) developing a new drawing starting from parallel lines. An example of a figural TTCT task might involve uniquely finishing a partially drawn image, with evaluations based on the aforementioned criteria (Rominger et al., 2018).

The TTCT includes a range of real-world reflective activities that encourage diverse thinking styles, essential for daily life and professional tasks. The TTCT assesses abilities in Questioning, Hypothesizing Causes and Effects, and Product Enhancement, each offering insights into an individual’s universal creative potential and originality (Boden, 2004; Runco and Jaeger, 2012; Sternberg, 2020). It acts like a comprehensive test battery, evaluating multiple facets of creativity’s complex nature (Guzik et al., 2023).

There are also other creativity tests such as Remote Associates Test (RAT), Runco Creativity Assessment Battery (rCAB), and Consensual Assessment Technique (CAT). TTCT is valued for its extensive historical database of human responses, which serves as a benchmark for comparison, owing to the consistent demographic profile of participants over many years and the systematic gathering of responses for evaluation (Kaufman et al., 2008). The Alternate Uses Task (AUT) and the Remote Associates Test (RAT) are appreciated for their straightforward administration, scoring, and analysis. The Creative Achievement Test (CAT) is notable for its adaptability to specific fields, made possible by employing a panel of experts in relevant domains to assess creative works. Consequently, the CAT is particularly suited for evaluating creative outputs in historical contexts or significant “Big-C” creativity (Kaufman et al., 2010). In contrast, the AUT and TTCT are more relevant for examining creativity in everyday, psychological, and professional contexts. As such, AUT and TTCT tests will establish a solid baseline for more complex design creativity studies employing more realistic design problems.

### 3.4 EEG recording and analysis: methods and algorithms

Electroencephalogram (EEG) signal analysis is a crucial component in the study of creativity whereby brain behavior associated with creativity tasks can be explored. Due to its advantages, EEG has emerged as one of the most suitable neuroimaging techniques for investigating brain activity during creativity tasks. Its affordability and suitability for studies involving physical movement, ease of recording and usage, and notably high temporal resolution make EEG a preferred choice in creativity research.

The dynamics during creative tasks are complex, nonlinear, and self-organized (Nguyen and Zeng, 2012). It can thus be assumed that the brain could exhibits the similar characteristics, which shall be reflected in EEG signals. Capturing these complex and nonlinear patterns of brain behavior can be challenging for other neuroimaging methods (Soroush et al., 2018).

#### 3.4.1 Preprocessing: artifact removal

In design creativity studies utilizing EEG, the susceptibility of EEG signals to noise and artifacts is a significant concern due to the accompanying physical movements inherent in these tasks. Consequently, EEG preprocessing becomes indispensable in ensuring data quality and reliability. Unfortunately, not all the included studies in this review have clearly explained their pre-processing and artifact removal approaches. There also exist some well-known preprocessing pipelines such as HAPPE (Gabard-Durnam et al., 2018) which (in contrast to their high efficiency) have been rarely used in design creativity neurocognition (Jia et al., 2021; Jia and Zeng, 2021). The included papers in our analysis have introduced various preprocessing methods, including wavelet analysis, frequency-based filtering, and independent component analysis (ICA) (Beaty et al., 2017; Fink et al., 2018; Lou et al., 2020). The primary objective of preprocessing remains consistent: to obtain high-quality EEG data devoid of noise or artifacts while minimizing information loss. Achieving this goal is crucial for the accurate interpretation and analysis of EEG signals in design creativity research.

#### 3.4.2 Preprocessing: segmentation

Design creativity studies often encompass a multitude of cognitive tasks occurring simultaneously or sequentially, rendering them ill-defined and unstructured. This complexity leads to the generation of unstructured EEG data, posing a challenge for subsequent analysis (Zhao et al., 2020). Therefore, segmentation methods play a crucial role in classifying recorded EEG signals into distinct cognitive tasks, such as idea generation, idea evolution, and idea evaluation.

Several segmentation methods have been adopted, including the ones relying on Task-Related Potential (TRP) analysis and microstate analysis, followed by clustering techniques like K-means clustering (Nguyen and Zeng, 2014a; Nguyen et al., 2019; Zhao et al., 2020; Jia et al., 2021; Jia and Zeng, 2021; Rominger et al., 2022b). These methods aid in organizing EEG data into meaningful segments corresponding to different phases of the design creativity process, facilitating more targeted and insightful analysis. In addition, they provide possibilities to look into a more comprehensive list of design-related cognitions implied in but not intended by conventional AUT and TTCT experiments.

While there are some uniform segmentation methods (such as the ones based on TRP) employing frequency-based methods. Nguyen et al. (2019) introduced a fully automatic dynamic method based on microstate analysis. Since then, microstate analysis has been used in several studies to categorize the EEG dynamics in design creativity tasks (Jia et al., 2021; Jia and Zeng, 2021). Microstate analysis provides a novel method for EEG-based design creativity studies with the capabilities of high temporal resolution and topography results (Yuan et al., 2012; Custo et al., 2017; Jia et al., 2021; Jia and Zeng, 2021).

#### 3.4.3 Feature extraction

The EEG data, after undergoing preprocessing, is directed to feature extraction, where relevant attributes are extracted to delve deeper into EEG dynamics and brain activity. These extracted features serve as the basis for conducting statistical analyses or employing machine learning algorithms.

In our review of the literature, we found that EEG frequency, time, and time-frequency analyses are the most commonly employed methods among the papers we considered. Specifically, the EEG alpha, beta, and gamma bands are often highlighted as critical indicators for studying brain dynamics in creativity and design creativity. Significant variations in the EEG bands have been observed during different stages of design creation tasks, including idea generation, idea evaluation, and idea elaboration (Nguyen and Zeng, 2010; Liu et al., 2016; Rominger et al., 2019; Giannopulu et al., 2022; Lukačević et al., 2023; Mazza et al., 2023). For instance, the very first creativity studies used EEG alpha asymmetry to explore the relationship between creativity and left-hemisphere and right-hemisphere brain activity (Martindale and Mines, 1975; Martindale and Hasenfus, 1978; Martindale et al., 1984). Other studies divided the EEG alpha band into lower (8–10 Hz) and upper alpha (10–13 Hz) and concluded that low alpha is more significant compared to the high EEG alpha band. Although the alpha band has been extensively explored by previous studies, several studies have also analyzed other EEG sub-bands such as beta, gamma, and delta and later concluded that these sub-bands are also significantly associated with creativity mechanisms, and can explain the differences between genders in different creativity experiments (Razumnikova, 2004; Volf et al., 2010; Nair et al., 2020; Vieira et al., 2022a).

Several studies have utilized Task-related power changes (TRP) to compare the EEG dynamics in different creativity tasks. TRP analysis is a high-temporal resolution method used to examine changes in brain activity associated with specific tasks or cognitive processes. In TRP analysis, the power of EEG signals, typically measured in terms of frequency bands (like alpha, beta, theta, etc.), is analyzed to identify how brain activity varies during the performance of a task compared to baseline or resting states. This method is particularly useful for understanding the dynamics of brain function as it allows researchers to pinpoint which areas of the brain are more active or less active during specific cognitive or motor tasks (Rominger et al., 2022b; Gubler et al., 2023). Reportedly, TRP has wide usage in EEG-based design creativity studies (Jia et al., 2021; Jia and Zeng, 2021; Gubler et al., 2022).

Event-related synchronization (ERS) and de-synchronization (ERD) have also been reported to be effective in creativity studies (Wang et al., 2017). ERD refers to a decrease in EEG power (in a specific frequency band) compared to a baseline state. The reduction in alpha power, for instance, is often interpreted as an increase in cortical activity. Conversely, ERS denotes an increase in EEG power. The increase in alpha power, for example, is associated with a relative decrease in cortical activity (Doppelmayr et al., 2002; Babiloni et al., 2014). Researchers have concluded that these two indicators play a pivotal role in creativity studies as they are significantly correlated with brain dynamics during creativity tasks (Srinivasan, 2007; Babiloni et al., 2014; Fink and Benedek, 2014).

Brain functional connectivity analysis, EEG source localization, brain topography maps, and event-related potentials analysis are other EEG processing methods which have been employed in a few studies (Srinivasan, 2007; Dietrich and Kanso, 2010; Giannopulu et al., 2022; Kuznetsov et al., 2023). Considering that these methods have not been employed in several studies and with respect to their potential to provide insight into brain activity in transient modes or the correlations between the brain lobes, future studies are suggested to utilize such methods.

#### 3.4.4 Data analysis and knowledge extraction

What was mentioned indicates that EEG frequency analysis is an effective approach for examining brain behavior in creativity and design creativity processes (Fink and Neubauer, 2006; Nguyen and Zeng, 2010; Benedek et al., 2011, 2014; Wang et al., 2017; Rominger et al., 2018; Vieira et al., 2022b). Analyzing EEG channels in the time or frequency domains across various creativity tasks helps identify key channels contributing to these experiments. TRP and ERD/ERS are well-known EEG analysis methods widely applied in the included studies. Some studies have used other EEG sub-bands such as delta or gamma (Boot et al., 2017; Stevens and Zabelina, 2020; Mazza et al., 2023). Besides these methods, other studies have utilized EEG connectivity and produced brain topography maps to explore different stages of design creativity. The final stage of EEG-based research involves statistical analysis and classification.

In statistical analysis, researchers examine EEG characteristics like power or alpha band amplitude to determine if there are notable differences during creativity tasks. Comparisons are made across different brain lobes and participants to identify which brain regions are more active during various stages of creativity. Techniques such as TRP, ERD, and ERS are scrutinized using statistical hypothesis testing to see if brain dynamics vary among participants or across different creativity tasks. Additionally, the relationship between EEG features and creativity scores is explored. For instance, researchers might investigate whether there is a link between EEG alpha power and creativity scores like originality and fluency. These statistical analyses can be conducted through either temporal or frequency EEG data.

In the classification phase, EEG data are classified according to different cognitive states of the brain. For example, EEG recordings might be classified based on the stages of creativity tasks, such as idea generation and idea evolution (Hu et al., 2017; Stevens and Zabelina, 2020; Lloyd-Cox et al., 2022; Ahad et al., 2023; Şekerci et al., 2024). Except for a few studies which employed machine learning, other studies targeted EEG analysis and statistical methods. In these studies, the main objective is reported to be the classification of designers’ cognitive states, their emotional states, or the level of their creativity. In the included papers, traditional classifiers such as support vector machines and k-nearest neighbor have been employed. Modern deep learning approaches can be used in future studies to extract the hidden valuable information of EEG in design creativity states (Jia, 2021). In open-ended loosely controlled creativity studies, where the phases of creativity are not clearly defined, clustering techniques are employed to categorize or segment EEG time intervals according to the corresponding creativity tasks (Jia et al., 2021; Jia and Zeng, 2021). While loosely controlled design creativity studies results in more reliable and natural outcomes compared to strictly controlled ones, analyzing EEG signals in loosely controlled experiments is challenging as the recorded signals are not structured. Clustering methods are applied to microstate analysis to segment EEG signals into pre-defined states and have structured blocks that may align with certain cognitive functions (Nguyen et al., 2019; Jia et al., 2021; Jia and Zeng, 2021). Therefore, statistical analysis, classification, and clustering form the core methods of data analysis in studies of creativity.

Table 2 represents EEG-based design studies with details about the number of participants, probable psychometric tests, experiment protocol, EEG analysis methods, and main findings. These studies are reported in this paper to highlight some of the differences between creativity and design creativity.

In addition to the studies reported in Table 2, previous reviews and studies (Srinivasan, 2007; Nguyen and Zeng, 2010; Lazar, 2018; Chrysikou and Gero, 2020; Hu and Shepley, 2022; Kim et al., 2022; Balters et al., 2023) can be found, which comprehensively reported approaches in design creativity neurocognition. Moreover, neurophysiological studies in design creativity are not limited to EEG or the components in Table 2. For instance, in Liu et al. (2014), EEG, heart rate (HR), and galvanic skin response (GSR) was used to detect the designer’s emotions in computer-aided design tasks. They determined the emotional states of CAD design tasks by processing CAD operators’ physiological signals and a fuzzy logic model. Aiello (2022) investigated the effects of external factors (such as light) and human ones on design processes, which also explored the association between the behavioral and neurophysiological responses in design creativity experiments. They employed ANOVA tests and found a significant correlation between neurophysiological recordings and daytime, participants’ stress, and their performance in terms of novelty and quality. They also recognized different patterns of brain dynamics corresponding to different kinds of performance measures. Montagna et al. (Montagna and Candusso, n.d.; Montagna and Laspia, 2018) analyzed brain behavior during the creative ideation process in the earliest phases of product development. In addition to EEG, they employed eye tracking to analyze the correlations between brain responses and eye movements. They utilized statistical analysis to recognize significant differences in brain hemispheres and lobes with respect to participants’ background, academic degree, and gender during the two modes of divergent and convergent thinking. Although some of their results are not consistent with those from the literature, these experiments shed light on the experiment design and provide insights and a framework for future experiments.

## 4 Discussion

In the present paper, we reviewed EEG-based design creativity studies in terms of their main components such as participants, psychometrics, and creativity tasks. Numerous studies have delved into brain activities associated with design creativity tasks, examined from various angles. While Table 1 showcases studies centered on the Alternate Uses Test (AUT), and the Torrance Tests of Creative Thinking (TTCT), Table 2 summarizes the EEG-based studies on design and design creativity-related tasks. In this section, we are going to discuss the impact of some most important factors including participants, experiment design, and EEG recording and processing on EEG-based design creativity studies. Research gaps and open questions are thus presented based on the discussion.

### 4.1 Participants

#### 4.1.1 Psychometrics: do we have a population that we wished for?

Psychometric testing is crucial for participant selection, with participant screening often based merely on self-reported information or based on their educational background. Examining Tables 1, 2 reveals that psychometrics are not frequently utilized in design creativity studies, indicating a notable gap in these investigations. Future research should consider establishing a standard set of psychometric tests to create comprehensive participant profiles, particularly focusing on intellectual capabilities (Jauk et al., 2015; Ueno et al., 2015; Razumnikova, 2022). Taking a look at the studies which employed psychometrics, it could be inferred that there is a correlation between cognitive abilities such as intelligence and creativity (Arden et al., 2010; Jung and Haier, 2013). The few psychometric tests employed primarily focus on determining and providing a cognitive profile, encompassing factors such as mood, stress, IQ, anxiety, memory, and intelligence. Notably, intelligence-related assessments are more commonly used compared to other tests. These psychometrics are subject to social masking according to which there is the possibility of unreliable self-report psychometrics being recorded in the experiments. These results might yield less accurate findings.

#### 4.1.2 Sample size and participants’ characteristics

Participant numbers in these studies vary widely, indicating a broad spectrum of sample sizes in this research area. The populations in the studies varied in size, with most having around 40 participants, predominantly students. In the design of experiments, it is important to highlight that the sample size in the selected studies had a mean of 43.76 and a standard deviation of 20.50. It is worth noting that while some studies employed specific experimental designs to determine sample size, many did not have clear and specific criteria for sample size determination, leaving the ideal sample size in such studies an open question. Any studies determine their sample sizes using G* power (Erdfelder et al., 1996; Faul et al., 2007), a prevalent tool for power analysis in social and behavioral research.

Initial investigations typically involved healthy adults to more thoroughly understand creativity’s underlying mechanisms. These foundational studies, conducted under optimal conditions, aimed to capture the essence of brain behavior during creative tasks. A handful of studies (Ayoobi et al., 2022; Gubler et al., 2022, 2023) have begun exploring creativity in the context of chronic pain or multiple sclerosis, but broader participant diversity remains an area for further research. Additionally, not all studies provided information on the ages of their participants. There is a noticeable gap in research involving older adults or those with health conditions, suggesting an area ripe for future exploration. Diversity in participant backgrounds, such as varying academic disciplines, could offer richer insights, given creativity’s multifaceted nature and its link to individual skills, affect, and perceived workload (Yang et al., 2022). For instance, the creative approaches of students with engineering thinking might differ significantly from those with art thinking.

Gender was not examined in most included studies. There are just a few studies analyzing the effects of gender on creativity and design creativity (Razumnikova, 2004; Volf et al., 2010; Vieira et al., 2020b, 2022a; Gubler et al., 2022). There is a notable need for further investigation to fully understand the impact of gender on the brain dynamics of design creativity.

### 4.2 Experiment design

While the Alternate Uses Test (AUT) and the Torrance Tests of Creative Thinking (TTCT) are commonly used in creativity research, other tasks like the Remote Associate Task are also prevalent (Schuler et al., 2019; Zhang et al., 2020). AUT and figural TTCT are particularly favored in design creativity experiments for their compatibility with design tasks, surpassing verbal or other creativity tasks in applicability (Boot et al., 2017). When considering the creativity tasks in the studies, it is notable that the AUT is more frequently utilized than TTCT, owing to its simplicity and ease of quantifying creativity scores. In contrast, TTCT often requires subjective assessments and expert ratings for scoring (Rogers et al., 2023). However, both TTCT and AUT have undergone modifications in several studies to investigate their potential characteristics further (Nguyen and Zeng, 2014a).

While the majority of studies have adhered to strictly controlled frameworks for their experiments, two studies (Nguyen and Zeng, 2017; Nguyen et al., 2019; Jia, 2021; Jia et al., 2021) have adopted novel, loosely controlled approaches, which reportedly yield more natural and reliable results compared to the strictly controlled ones. The rigidity from strictly controlled creativity experiments can exert additional cognitive stress on participants, potentially impacting experimental outcomes. In contrast, the loosely controlled experiments are characterized as self-paced and open-ended, allowing participants ample time to comprehend the design problem, generate ideas, evaluate them, and iterate upon them as needed. Recent behavioral and theoretical research suggests that creativity is better explored within a loosely controlled framework, where sufficient flexibility and freedom are essential. This approach, which contrasts with the highly regulated nature of traditional creativity studies, aims to capture the unpredictable elements of design activities (Zhao et al., 2020). Loosely controlled design studies offer a more realistic portrayal of the actual design process. In these settings, participants enjoy the liberty to develop ideas at their own pace, reflecting true design practices (Jia, 2021). The flexibility in such experiments allows for a broader range of scenarios and outcomes, depending on the complexity and the designers’ understanding of the tests and processes. Prior research has confirmed the effectiveness of this approach, examining its validity from both neuropsychological and design perspectives. Despite their less rigid structure, these loosely controlled experiments are valid and consistent with previous studies. Loosely controlled creativity experiments allow researchers to engage with the nonlinear, ill-defined, open-ended, and intricate nature of creativity tasks. However, it is important to note that data collection and processing can pose challenges in loosely controlled experiments due to the resulting unstructured data. These challenges can be handled through machine learning and signal processing methods (Zhao et al., 2020). For further details regarding the loosely controlled experiments, readers can refer to the provided references (Zhao et al., 2020; Jia et al., 2021; Jia and Zeng, 2021; Zangeneh Soroush et al., 2024).

Participants are affected by external or internal sources during the experiments. Participants are asked not to have caffeine, alcohol, or other stimulating beverages. The influence of stimulants like caffeine, alcohol, and other substances on creative brain dynamics is another under-researched area. While some studies have investigated the impact of cognitive and affective stimulation on creativity [such as pain (Gubler et al., 2022, 2023)], more extensive research is needed. The study concerning environmental factors like temperature, humidity, and lighting, has been noted to significantly influence creativity (Kimura et al., 2023; Lee and Lee, 2023). Investigating these environmental aspects could lead to more conclusive findings. Understanding these variables related to participants and their surroundings will enable more holistic and comprehensive creativity studies.

### 4.3 EEG

#### 4.3.1 Advantages and disadvantages of EEG being used in design creativity experiments

As previously discussed and generally known in the neuroscience research community, EEG stands out as a simple and cost-effective biosignal with high temporal resolution, facilitating the exploration of microseconds of brain dynamics and providing detailed insights into neural activity, which was summarized in Balters and Steinert (2017) and Soroush et al. (2018). However, despite its advantages in creativity experiments, EEG recording is prone to high levels of noise and artifacts due to its low amplitude and bandwidth (Zangeneh Soroush et al., 2022). The inclusion of physical movements in design creativity experiments further increases the likelihood of artifacts such as movement and electrode replacement artifacts. Additionally, it is essential to acknowledge that EEG does have limitations, including relatively low spatial resolution. It also provides less information regarding brain behavior compared to other methods such as fMRI which provides detailed spatial brain activity.

#### 4.3.2 EEG processing and data analysis

In design creativity experiments, EEG preprocessing is an inseparable phase ensuring the quality of EEG data in design creativity experiments. Widely employed artifact removal methods include frequency-based filters and independent component analysis. Unfortunately, not all studies provide a detailed description of their artifact removal procedures (Zangeneh Soroush et al., 2022), compromising the reproducibility of the findings. Moreover, while there are standard evaluation metrics for assessing the quality of preprocessed EEG data, these metrics are often overlooked or not discussed in the included papers. It is essential to note that EEG preprocessing extends beyond artifact removal to include the segmentation of unstructured EEG data into well-defined structured EEG windows each of which corresponds to a specific cognitive task. This presents a challenge, particularly in loosely controlled experiments where the cognitive activities of designers during drawing tasks may not be clearly delineated since design tasks are recursive, nonlinear, self-paced, and complex, further complicating the segmentation process (Nguyen and Zeng, 2012; Yang et al., 2022).

EEG analysis methods in creativity research predominantly utilize frequency-based analysis, with the alpha band (particularly the upper alpha band, 10–13 Hz) being a key focus due to its effectiveness in capturing various phases of creativity, including divergent and convergent thinking. Across studies, a consistent pattern of decreases in EEG power during design creativity compared to rest has been observed in the low-frequency delta and theta bands, as well as in the lower and upper alpha bands in bilateral frontal, central, and occipital brain regions (Fink and Benedek, 2014, 2021). This phenomenon, known as task-related desynchronization (TRD), is a common finding in EEG analysis during creativity tasks (Jausovec and Jausovec, 2000; Pidgeon et al., 2016). A recurrent observation in numerous studies is the link between alpha band activity and creative cognition, particularly original idea generation and divergent thinking. Alpha synchronization, especially in the right hemisphere and frontal regions, is commonly associated with creative tasks and the generation of original ideas (Rominger et al., 2022a). Task-Related Power (TRP) analysis in the alpha band is widely used to decipher creativity-related brain activities. Creativity tasks typically result in increased alpha power, with more innovative responses correlating with stronger alpha synchronization in the posterior cortices. The TRP dynamics, marked by an initial rise, subsequent fall, and a final increase in alpha power, reflect the cognitive processes underlying creative ideation (Rominger et al., 2018). Creativity is influenced by both cognitive processes and affective states, with studies showing that cognitive and affective interventions can enhance creative cognition through stronger prefrontal alpha activity. Different creative phases (e.g., idea generation, evolution, evaluation) exhibit unique EEG activity patterns. For instance, idea evolution is linked to a smaller decrease in lower alpha power, indicating varying attentional demands (Fink and Benedek, 2014, 2021; Rominger et al., 2019, 2022a; Jia and Zeng, 2021).

Hemispheric asymmetry plays a crucial role in creativity, with increased alpha power in the right hemisphere linked to the generation of more novel ideas. This asymmetry intensifies as the creative process unfolds. The frontal cortex, particularly through alpha synchronization, is frequently involved in creative cognition and idea evaluation, indicating a role in top-down control and internal attention (Benedek et al., 2014). The parietal cortex, especially the right parietal cortex, is significant for focused internal attention during creative tasks (Razumnikova, 2004; Benedek et al., 2011, 2014).

EEG phase locking is another frequently employed analysis method. Most studies have focused on EEG coherence, EEG power and frequency analysis, brain asymmetry methods (hemispheric lateralization), and EEG temporal methods (Rominger et al., 2020). However, creativity, being a higher-order, complex, nonlinear, and non-stationary cognitive task, suggests that linear and deterministic methods like frequency-based analysis might not fully capture its intricacies. This raises the possibility of incorporating alternative, specifically nonlinear EEG processing methods, which, to our knowledge, have been sparingly used in creativity research (Stevens and Zabelina, 2020; Jia and Zeng, 2021). Additional analyses such as wavelet analysis, brain source separation, and source localization hold promise for future research endeavors in this domain.

As mentioned in the previous section, most studies have considered participants without their cognitive profile and characteristics. In addition, the included studies have chosen two main approaches including traditional statistical analysis and machine learning methods (Goel, 2014; Stevens and Zabelina, 2020; Fink and Benedek, 2021). It should be noted that almost all of the included studies have employed the traditional statistical methods to examine their hypotheses or explore the differences between participants performing creativity tasks (Fink and Benedek, 2014, 2021; Rominger et al., 2019, 2022a; Stevens and Zabelina, 2020; Jia and Zeng, 2021).

Individual differences, such as intelligence, personality traits, and humor comprehension, also affect EEG patterns during creative tasks. For example, individuals with higher monitoring skills and creative potential exhibit distinct alpha power changes during creative ideation and evaluation (Perchtold-Stefan et al., 2020). The diversity in creativity tasks (e.g., AUT, TTCT, verbal tasks) and EEG analysis methods (e.g., ERD/ERS, TRP, phase locking) used in studies highlights the methodological variety in this field, emphasizing the complexity of creativity research and the necessity for multiple approaches to fully grasp its neurocognitive mechanisms (Goel, 2014; Gero and Milovanovic, 2020; Rominger et al., 2020; Fink and Benedek, 2021; Jia and Zeng, 2021).

In statistical analysis, studies often assess the differences in extracted features across different categories. For instance, in a study (Gopan et al., 2022), various features, including nonlinear and temporal features, are extracted from single-channel EEG data to evaluate levels of Visual Creativity during sketching tasks. This involves comparing different groups within the experimental population based on specific features. Notably, the traditional statistical analyses not only provide insights into differences between experimental groups but also offer valuable information for machine learning methods (Stevens and Zabelina, 2020). In another study (Gubler et al., 2023), researchers conducted statistical analysis on frequency-based features to explore the impact of experimentally induced pain on creative ideation among female participants using an adaptation of the Alternate Uses Task (AUT). The analysis involved examining EEG features across channels and brain hemispheres under pain and pain-free conditions. Similarly, in another study (Benedek et al., 2014), researchers conducted statistical analysis on EEG alpha power to investigate the functional significance of alpha power increases in the right parietal cortex, which reflects focused internal attention. They found that the Alternate Uses Task (AUT) inherently relies on internal attention (sensory-independence). Specifically, enforcing internal attention led to increased alpha power only in tasks requiring sensory intake but not in tasks requiring sensory independence. Moreover, sensory-independent tasks generally exhibited higher task-related alpha power levels than sensory intake tasks across both experimental conditions (Benedek et al., 2011, 2014).

Although most studies have employed statistical measures and analyses to investigate brain dynamics in a limited number of participants, there is a considerable lack of within-subjects and between-subjects analyses (Rominger et al., 2022b). There exist several studies which differentiate the brain dynamics of expert and novice designers or engineering students in different fields (Vieira et al., 2020c,d); however, more investigations with a larger number of participants are required.

While statistical approaches are commonly employed in EEG-based design creativity studies, there is a notable absence of machine learning methods within this domain. Among the included studies, only one (Gopan et al., 2022) utilized machine learning techniques. In this study, statistical and nonlinear features were extracted from preprocessed EEG signals to classify EEG data into predefined cognitive tasks based on EEG characteristics. The study employed machine learning algorithms such as Long Short-Term Memory (LSTM), Support Vector Machines (SVM), and k-Nearest Neighbor (KNN) to classify EEG samples. These methods were utilized to enhance the understanding of the relationship between EEG signals and cognitive tasks, offering a promising avenue for further exploration in EEG-based design creativity research (Stevens and Zabelina, 2020).

### 4.4 Research gaps and open questions

In this review, we aimed to empower readers to decide on experiments, EEG markers, feature extraction algorithms, and processing methods based on their study objectives, requirements, and limitations. However, it is essential to acknowledge that this review, while valuable in exploring EEG-based creativity and design creativity, has certain limitations which are summarized below:

1. Our review focuses on just the neuroscientific aspects of prior creativity and design creativity studies. Design methodologies and creativity models should be reviewed in other studies.
2. Included studies have employed only a limited number of adult participants with no mental or physical disorder.
3. Most studies have utilized fNIRS or EEG as they are more suitable for design creativity experiments, but we only focused on EEG based studies.

According to what was discussed above, it is obvious that EEG-based design creativity studies have been quite recently introduced to the field of design. This indicates that research gaps and open questions should be addressed for future studies. The following provides ten open questions we extracted from this review.

1. What constitutes an optimal protocol for participant selection, creativity task design, and procedural guidelines in EEG-based design creativity research?
2. How can we reconcile inconsistencies arising from variations in creativity tests and procedures across different studies? Furthermore, how can we address disparities between findings in EEG and fMRI studies?
3. What notable disparities exist in brain dynamics when comparing different creativity tests within the realm of design creativity?
4. In what ways can additional physiological markers, such as ECG and eye tracking, contribute to understanding neurocognition in design creativity?
5. How can alternative EEG processing methods beyond frequency-based analysis enhance the study of brain behavior during design creativity tasks?
6. What strategies can be employed to integrate combinational methods like EEG-fMRI to investigate design creativity?
7. How can the utilization of advanced wearable recording systems facilitate the implementation of more naturalistic and ecologically valid design creativity experiments?
8. What are the most effective approaches for transforming unstructured data into organized formats in loosely controlled creativity experiments?
9. What neural mechanisms are associated with design creativity in various mental and physical disorders?
10. In what ways can the application of advanced EEG processing methods offer deeper insights into the neurocognitive aspects of design creativity?

## 5 Conclusion

Design creativity stands as one of the most intricate high-order cognitive tasks, encompassing both mental and physical activities. It is a domain where design and creativity are intertwined, each representing a complex cognitive process. The human brain, an immensely sophisticated biological system, undergoes numerous intricate dynamics to facilitate creative abilities. The evolution of neuroimaging techniques, computational technologies, and machine learning has now enabled us to delve deeper into the brain behavior in design creativity tasks.

This literature review aims to scrutinize and highlight pivotal, and foundational research in this area. Our goal is to provide essential, comprehensive, and practical insights for future investigators in this field. We employed the snowball search method to reach the final set of papers which met our inclusion criteria. In this review, more than 1,500 studies were monitored and assessed as EEG-based creativity and design creativity studies. We reviewed over 120 studies with respect to their experimental details including participants, (design) creativity tasks, EEG analyses methods, and their main findings. Our review reports the most important experimental details of EEG-based studies and it also highlights research gaps, potential future trends, and promising avenues for future investigations.

## Author contributions

MZ: Formal analysis, Investigation, Writing – original draft, Writing – review & editing. YZ: Conceptualization, Funding acquisition, Methodology, Project administration, Resources, Supervision, Writing – review & editing.
